# Hyperpolarized mitochondria accumulate in *Drosophila* Hipk-overexpressing cells to drive tumor-like growth

**DOI:** 10.1242/jcs.250944

**Published:** 2020-12-09

**Authors:** Kenneth Kin Lam Wong, Jenny Zhe Liao, Claire R. Y. Shih, Nicholas Harden, Esther M. Verheyen

**Affiliations:** 1 Department of Molecular Biology and Biochemistry, Simon Fraser University, Burnaby, British Columbia, V5A 1S6, Canada; 2 Centre for Cell Biology, Development and Disease, Simon Fraser University, Burnaby, British Columbia, V5A 1S6, Canada

**Keywords:** *Drosophila*, Hipk, Myc, ROS, Energetics, Mitochondria

## Abstract

Both functional and dysfunctional mitochondria are known to underlie tumor progression. Here, we establish use of the proto-oncogene *Drosophila* Homeodomain-interacting protein kinase (Hipk) as a new tool to address this paradox. We find that, in Hipk-overexpressing tumor-like cells, mitochondria accumulate and switch from fragmented to highly fused interconnected morphologies. Moreover, elevated Hipk promotes mitochondrial membrane hyperpolarization. These mitochondrial changes are at least in part driven by the upregulation of Myc. Furthermore, we show that the altered mitochondrial energetics, but not morphology, is required for Hipk-induced tumor-like growth, because knockdown of *pdsw* (also known as *nd-pdsw*; *NDUFB10* in mammals; a Complex I subunit) abrogates the growth. Knockdown of *ATPsynβ* (a Complex V subunit), which produces higher levels of reactive oxygen species (ROS) than *pdsw* knockdown, instead synergizes with Hipk to potentiate JNK activation and the downstream induction of matrix metalloproteinases. Accordingly, *ATPsynβ* knockdown suppresses Hipk-induced tumor-like growth only when ROS scavengers are co-expressed. Together, our work presents an *in vivo* tumor model featuring the accumulation of hyperfused and hyperpolarized mitochondria, and reveals respiratory complex subunit-dependent opposing effects on tumorigenic outcomes.

This article has an associated First Person interview with the first author of the paper.

## Abstract (precis)

**Summary:** Altered mitochondrial energetics plays a more decisive role than morphology in determining tumor-like growth in a fly tumor model caused by elevated Homeodomain-interacting protein kinase.

## INTRODUCTION

In the 1920s, Otto Warburg observed that cancer cells take up glucose and produce lactate vigorously even in the presence of oxygen, a phenomenon now termed the Warburg effect or aerobic glycolysis (Warburg et al., 1927). Warburg hypothesized that mitochondrial dysfunction is a cause of aerobic glycolysis and cancers (Warburg, 1956), which remains highly debated today. In support of this hypothesis, succinate dehydrogenase (SDH) and fumarase, which are mitochondrial enzymes of the tricarboxylic acid (TCA) cycle, were identified as tumor suppressors (Gottlieb and Tomlinson, 2005; King et al., 2006). Mutations in SDH or fumarase in cancer lead to stabilization of hypoxia-inducible factor 1-alpha (HIF1α) and thus activate a pseudo-hypoxic response (Isaacs et al., 2005; Selak et al., 2005). Meanwhile, a growing body of evidence contradicts Warburg's hypothesis. For example, despite high glycolytic rates, many cancer cells generate most ATP through oxidative phosphorylation (reviewed by Zu and Guppy, 2004). Overall, cancer mitochondrial metabolism is more heterogeneous than previously thought, and there is a need for an effective model system to aid in devising therapeutic strategies targeting mitochondria.

*Drosophila melanogaster* (fruit fly) has been extensively used to model human cancers largely due to the high conservation between human and fly genes (Sonoshita and Cagan, 2017). Interestingly, most, if not all, previously described fly tumor models display features of mitochondrial dysfunction, including reduced respiratory complex activity and massive generation of reactive oxygen species (ROS). For example, the well-known *Ras^V12^ scribble^−/−^* tumors harbor damaged mitochondria and high levels of ROS (Katheder et al., 2017). In tumors with activated platelet-derived growth factor receptor or vascular endothelial growth factor receptor (Pvr), pyruvate dehydrogenase (PDH) is inactivated by PDH kinase, thus attenuating mitochondrial respiration and causing high ROS (Wang et al., 2016). Nonetheless, growing evidence points to the importance of functional mitochondria in fly tumorigenesis. For instance, inhibition of respiratory complex activity can suppress larval brain tumor growths caused by loss of *Brain tumor* (*Brat*) (Van Den Ameele and Brand, 2019). Also, blocking mitochondrial fusion reduces overgrowths induced by activated Yorkie (Yki; Yes-associated protein, YAP in vertebrates) (Nagaraj et al., 2012). However, the respiratory profiles of these tumor models have not been clearly characterized and, until now, a *Drosophila* tumor model with functional mitochondria has not been reported.

Homeodomain-interacting protein kinases (Hipk in fly, HIPK1-4 in vertebrates) are transcriptional co-regulators that interact with multiple cellular signaling pathways (Chen and Verheyen, 2012; Kim et al., 1998; Lee et al., 2009; Swarup and Verheyen, 2011; Tettweiler et al., 2019). Dysregulation of HIPKs has been implicated in certain diseases and cancers (reviewed by Blaquiere and Verheyen, 2017). For instance, elevation of HIPK2 (the most studied member of the family) is associated with the malignancy of pilocytic astrocytomas (Deshmukh et al., 2008), cervical carcinogenesis (Al-Beiti and Lu, 2008) and human papillomavirus-positive tonsillar squamous cell carcinomas (Kwon et al., 2017). In *Drosophila*, elevated Hipk induces neoplastic overgrowth, invasion-like behaviors and cellular changes reminiscent of an epithelial-to-mesenchymal transition, including induction of Matrix metalloproteinase 1 (MMP1) and loss of E-Cadherin (Blaquiere et al., 2018). Recently, we found that elevated Hipk promotes proto-oncogene Myc-driven aerobic glycolysis, which in turn functions to sustain the accumulation of Myc, forming a positive feedback loop to support tumor-like growth (Wong et al., 2019). Here, we continue characterizing the metabolic profile of Hipk tumor-like cells, with a particular focus on mitochondrial metabolism. Probing mitochondrial morphology, mass and membrane potential (Δψ_m_), we show that Hipk tumor-like cells abound with hyperfused and hyperpolarized mitochondria, and that this metabolic shift depends on Myc upregulation. Our work further identifies Pdsw (also known as ND-PDSW; NDUFB10 in mammals), a Complex I subunit, as a metabolic vulnerability in Hipk tumor-like cells as its inhibition abrogates growth without generating excessive amounts of ROS.

## RESULTS

### Elevated Hipk promotes mitochondrial fusion and mass

**Fig. 1.**
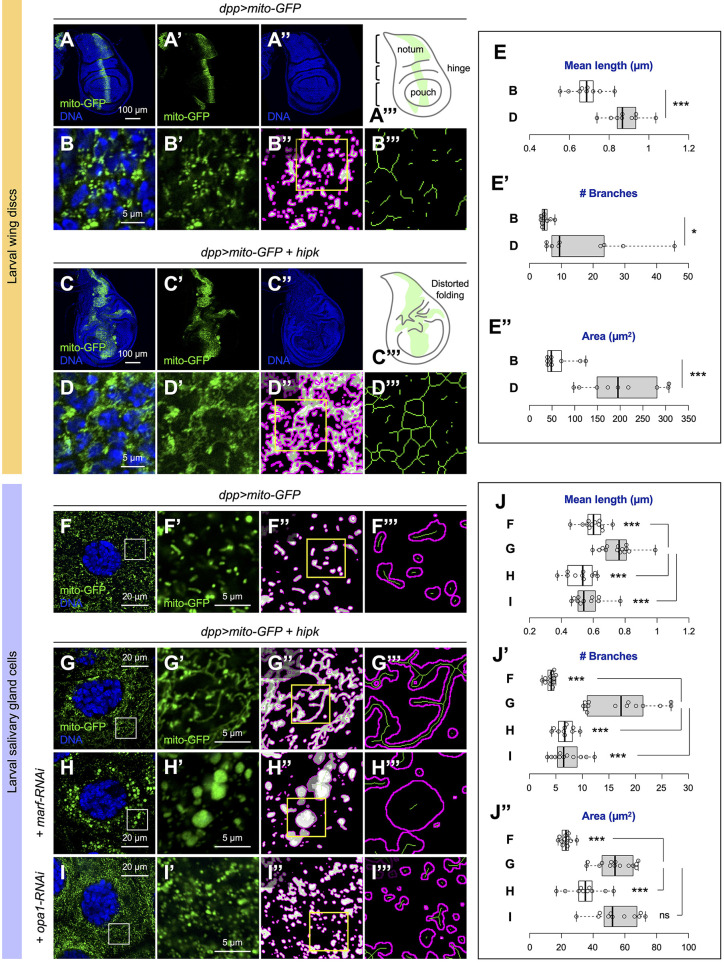
**Elevated Hipk promotes mitochondrial fusion and mass.** (A,B) Control (*dpp>mito-GFP*) wing disc expressing mito-GFP (marking mitochondria in green) under the control of *dpp-Gal4.* (A‴) Diagram of a control wing disc, which is mainly composed of three regions known as the notum, hinge and pouch. The transgene-expressing Dpp domain is shaded in light green. Mitochondria of control DP cells were labeled with mito-GFP (green; B,B′), and were processed by MiNA with mitochondrial outline shown in magenta and skeleton in green (B″,B‴). Yellow inset in B″ is magnified in B‴. (C,D) *hipk-*overexpressing (*dpp>mito-GFP+hipk*) wing disc expressing mito-GFP (in green). (C‴) Diagram of a *hipk-*overexpressing wing disc displaying distorted tissue morphology. Mitochondria of *hipk-*overexpressing DP cells were labeled with mito-GFP (green; D,D′), and were processed by MiNA with mitochondrial outline shown in magenta and skeleton in green (D″,D‴). Yellow inset in D″ is magnified in D‴. (E-E″) Box and whisker plots showing the mean mitochondrial length (E), the number of branches (E′) and the mitochondrial area (E″) in control (B; *dpp>mito-GFP*) and *hipk-*overexpressing (D; *dpp>mito-GFP+hipk*) wing discs. (F-I) Airyscan images of mitochondria (marked by mito-GFP in green) in larval salivary gland cells of the indicated genotypes: (F) *dpp>mito-GFP*, (G) *dpp>mito-GFP+hipk*, (H) *dpp>mito-GFP+hipk+marf-RNAi* and (I) *dpp>mito-GFP+hipk+opa1-RNAi*. Insets in F-I are magnified in F′-I′. Images F′-I′ were processed by MiNA, with mitochondrial outline shown in magenta and skeleton in green (F″-I″,F‴-I‴). Yellow insets in F″-I″ are magnified in F‴-I‴. (J-J″) Box and whisker plots showing the mean mitochondrial length (J), the number of branches (J′) and the mitochondrial area (J″) in salivary glands of the indicated genotypes. Letters F-I refer to the genotypes shown in panels F-I. DAPI staining for DNA (blue) shows the overall tissue morphologies and nuclei. *P*-values were calculated using unpaired two-tailed *t-*test: *P<0.05, ***P<0.001; ns, not significant.

Wing imaginal discs of *Drosophila* larvae, which are epithelial tissues that give rise to adult wing structures, are commonly used to model human carcinomas (Herranz et al., 2016). To generate the fly Hipk tumor model, we used the Gal4-UAS system (Brand and Perrimon, 1993) to induce *UAS-hipk* transgene overexpression under the control of *dpp-Gal4* driver in larval wing discs. Fluorescent proteins (e.g. mito-GFP, a mitochondrially targeted GFP) were co-expressed to mark the transgene-expressing cells. As previously reported (Blaquiere et al., 2018; Wong et al., 2019, 2020), in contrast to control wing discs (*dpp>mito-GFP*) (Fig. 1A), *hipk-*overexpressing discs (*dpp>mito-GFP+hipk*) manifested marked expansion of the GFP-positive area (the transgene-expressing Dpp domain) and distorted tissue morphology (as revealed by DNA staining) (Fig. 1C). Occasionally, some isolated GFP-positive *hipk-*overexpressing cells were found outside of the Dpp domain, indicative of cell invasion-like behavior (Fig. S1B, yellow arrowheads; compare with control in Fig. S1A).

In our earlier study, we showed that elevated Hipk increases glucose uptake and glycolytic flux (Wong et al., 2019). Given that mitochondrial metabolism can be influenced by alterations in energy demand and nutrient supply (Liesa and Shirihai, 2013; Spurlock et al., 2019), we were motivated to examine whether elevated Hipk leads to any changes in mitochondrial dynamics, abundance and energetics. We first examined mitochondrial morphology using mito-GFP (Pilling et al., 2006). In control wing disc cells, most of the mitochondria appeared as puncta around the nuclei (Fig. 1B,B′). In contrast, in Hipk tumor-like cells, patches of fused mitochondria accumulated around the nuclei (Fig. 1D,D′). Several image analysis tools such as MitoGraph (Viana et al., 2015), MiNA (Valente et al., 2017) and Mito-SinCe^2^ (Spurlock et al., 2019) have been developed for quantifying mitochondrial dynamics. In this study, we used the MiNA (Mitochondrial Network Analysis) toolset to generate skeleton images for quantitative analyses of mitochondrial morphology (Fig. 1B″,B‴,D″,D‴). The MiNA analysis revealed that elevation of Hipk was associated with significant increases in the mitochondrial length and the number of branches (Fig. 1E,E′). Using the mito-GFP-positive area to determine mitochondrial mass, we found that elevated Hipk led to a drastic increase in mitochondrial abundance (Fig. 1E″).

We also examined two other larval tissues, body wall muscle and salivary glands, because their large cell size facilitates imaging of mitochondria. In control muscle cells, where mito-GFP was expressed under a muscle-specific Gal4 driver *mef2* (*mef2>mito-GFP*), mitochondria were abundant, and they appeared rod-shaped and tubular (Fig. S2A). Upon *hipk* overexpression (*mef2>mito-GFP+hipk*), mitochondria were highly convoluted as indicated by a significant increase in the extent of branching (Fig. S2B,C). Elevated Hipk did not cause statistically significant changes in mitochondrial length or mass in muscle cells (Fig. S2C-C″), probably because the mitochondria in control cells were already fused and abundant.

In larval salivary glands, mitochondria appeared punctate in control cells (*dpp>mito-GFP*) (Fig. 1F), whereas elongated and branched mitochondria were enriched in *hipk-*overexpressing cells (*dpp>mito-GFP+hipk*) (Fig. 1G). Using the MiNA toolset, we found that elevation of Hipk caused significant increases in the mitochondrial length, branching and mass (Fig. 1J-J″), corroborating the results obtained from wing discs (Fig. 1A-E). Three-dimensional (3D) imaging acquired by Airyscan super-resolution microscopy revealed individual mitochondria of various sizes and shapes (including punctate, rod-like, round, network and irregular) in control salivary gland cells (Fig. S2D, Movie 1) in contrast to meshes of mitochondria with extensive, fine branches in *hipk*-overexpressing cells (Fig. S2E, Movie 2).

Mitochondrial morphology is highly dynamic and is determined by the balance of mitochondrial fusion and fission (van der Bliek et al., 2013). Mitofusins (Mfn 1-2 in mammals; Marf, Mitochondrial assembly regulatory factor, in *Drosophila*) and Opa1 (Optic atrophy protein 1) are dynamin-related GTPases that mediate the fusion of outer membrane and inner membrane, respectively. The mitochondrial fusion phenotype observed in *hipk-*overexpressing salivary gland cells (Fig. 1G) was reversed when *marf* was knocked down using RNA interference (RNAi), resulting in large, globular mitochondrial particles (Fig. 1H). Upon *opa1* knockdown, the mitochondria in *hipk*-overexpressing cells appeared as tiny individual puncta, exhibiting a robust fission phenotype (Fig. 1I). Quantitative analyses confirmed that knockdown of either *marf* or *opa1* led to decreases in mitochondrial length and branching in *hipk-*overexpressing cells (Fig. 1J). Thus, our data suggest that elevated Hipk increases mitochondrial mass and promotes the accumulation of long, tubular mitochondrial networks, especially in wing disc and salivary gland epithelial cells.

### Hipk tumor-like cells abound with hyperpolarized mitochondria

**Fig. 2.**
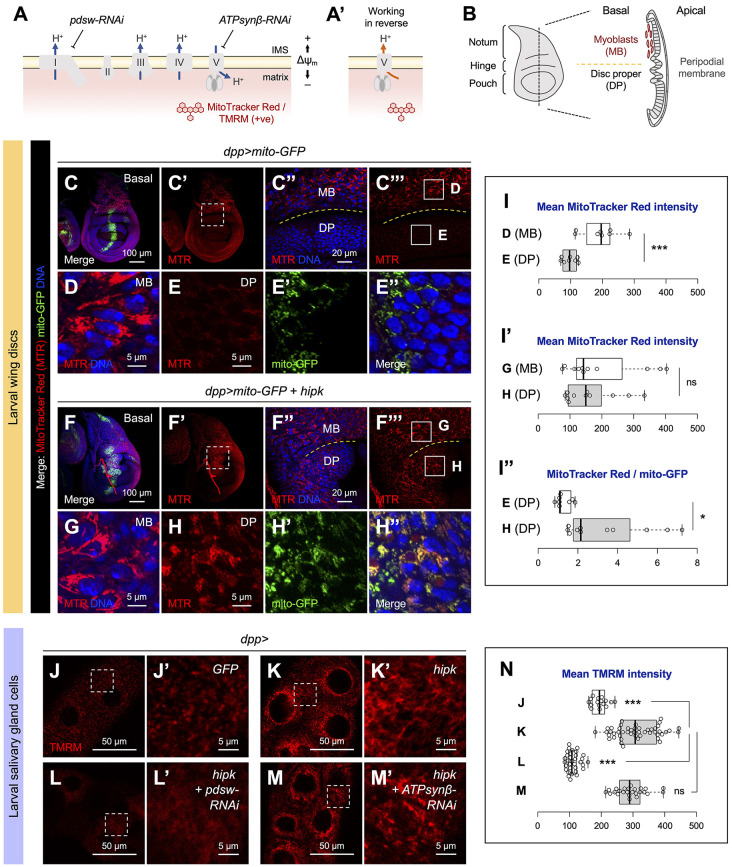
**Hipk tumor-like cells abound with hyperpolarized mitochondria.** (A,A′) Diagram depicting the mitochondrial complexes in the mitochondrial inner membrane. Complexes I-V are shown in gray. Blue arrows in A and orange arrow in A′ indicate proton transport. Electron transfer is not shown for simplicity. Cationic MitoTracker Red or TMRM (red) accumulates in the matrix when the membrane potential (Δψ_m_) is established. The activities of Complexes I and V can be genetically inhibited by *pdsw-RNAi* and *ATPsynβ-RNAi*, respectively. IMS, intermembrane space. In mitochondria where respiratory activity is compromised, Δψ_m_ can be generated by the reversal of Complex V function (A′). (B) Diagram showing a wild-type third instar larval wing disc. Left: During development, the wing disc is folded, giving rise to three well-defined regions that give rise to the adult structures, known as the notum, hinge and pouch. Right: Cross-sectional view of the wing disc showing the peripodial membrane on the apical side, the DP on the basal side and the attached myoblasts (MBs). (C-E) The basal side of a control wing disc (*dpp>mito-GFP*) stained with MitoTracker Red (MTR) (red). Mito-GFP (green) was expressed in the Dpp domain to label mitochondria. Inset (dashed line box) in C′ is magnified in C″ and C‴. The dashed lines in C″ and C‴ separate the MBs (above the line) from the adjacent DP cells (below the line). Insets (solid line boxes) in C‴ are magnified in D and E to show the MTR staining in MB cells (D) and in control DP cells (E), respectively. (F-H) The basal side of a *hipk-*overexpressing wing disc (*dpp>mito-GFP+hipk*) stained with MitoTracker Red (MTR) (red). Mito-GFP (green) was expressed in the Dpp domain to label mitochondria. Inset (dashed line box) in F′ is magnified in F″ and F‴. The dashed lines in F″ and F‴ separate the MBs (above the line) from the adjacent DP cells (below the line). Insets (solid line boxes) in F‴ are magnified in G and H to show the MTR staining in MB cells (G) and in *hipk-*overexpressing DP cells (H), respectively. (I) Box and whisker plots showing the mean MitoTracker Red intensities of MB and DP cells in control wing discs (I), of MB and DP cells in *hipk-*overexpressing wing discs (I′), and the ratio of MitoTracker Red to mito-GFP signal intensities of control and *hipk-*overexpressing DP cells (I″). Letters D, E, G and H refer to the cells of the indicated genotypes shown in panels D,E,G,H. (J-M) Incorporation of TMRM (red) in live control (*dpp>GFP*) (J), *hipk-*overexpressing (*dpp>hipk*) (K), *hipk* and *pdsw-RNAi* co-expressing (*dpp>hipk+pdsw-RNAi*) (L) and *hipk* and *ATPsynβ-RNAi* co-expressing (*dpp>hipk+ATPsynβ-RNAi*) salivary gland cells (M). Insets in J-M are magnified in J′-M′. (N) A box and whisker plot showing the mean TMRM fluorescence in live salivary gland cells of the indicated genotypes. Letters J-M refer to the genotypes shown in panels J-M. DNA (blue) was stained by DAPI. *P*-values were calculated using unpaired two-tailed *t-*test: *P<0.05, ***P<0.001; ns, not significant.

The Hipk-induced shift in mitochondrial morphology from fragmentation to fusion prompted us to ask whether mitochondrial energetics are affected. To assess the energetics, we stained cells with MitoTracker Red, a cationic red fluorescent probe for monitoring the mitochondrial membrane potential (Δψ_m_), which correlates with the respiratory capacity to generate ATP (Fig. 2A) (Pendergrass et al., 2004). In active mitochondria where a negative Δψ_m_ is established (membrane hyperpolarization), MitoTracker Red is retained in the mitochondrial matrix. In mitochondria where the membrane is depolarized, the dye does not accumulate in the matrix.

The larval wing disc is a sac-like structure composed of the squamous peripodial epithelium (sometimes called peripodial membrane) on the apical side connected to the disc proper (DP, also known as the columnar epithelium) on the basal side, which are separated by a lumen (Cohen, 1993) (Fig. 2B). In the MitoTracker Red staining experiments, we focused on the basal side because the transgene-expressing cells (DP cells) on this side were more exposed to the mitochondrial dye. In control wing discs (Fig. 2C-E), we observed that MitoTracker Red primarily accumulated in myoblasts (MB; the progenitors of adult flight muscles), and less MitoTracker Red was taken up by DP cells (Fig. 2C‴, compare inset D with inset E; Fig. 2D,E,I). Because the MB and DP cells were captured on the same focal plane (revealed by DNA staining), these cells were presumably exposed to MitoTracker Red to the same extent. The difference in the MitoTracker Red uptake of MB and DP cells suggests that these two cell types have different energetic states and/or mitochondrial mass.

Intriguingly, in *hipk-*overexpressing discs (Fig. 2F-H), both MB and Hipk tumor-like cells (the *hipk-*overexpressing DP cells) had comparably high MitoTracker Red signals (Fig. 2F‴, compare inset G with inset H; Fig. 2G,H,I′). Thus, using the MitoTracker Red intensity in myoblasts as an internal reference in each wing disc to ensure that the DP cells we captured had been exposed to similar amounts of MitoTracker Red, we found that *hipk-*overexpressing DP cells took up more MitoTracker Red than the control DP cells (compare Fig. 2H with Fig. 2E). Given that MitoTracker staining is a measure of both mitochondrial mass and Δψ_m_ and that we observed an increase in mitochondrial abundance upon elevation of Hipk, we determined Δψ_m_ using the ratio of MitoTracker Red to mito-GFP signal intensities and found that mitochondria in *hipk-*overexpressing cells were more polarized than those in control cells (Fig. 2I″). The MitoTracker Red staining patterns of wing discs along the z-axis are shown in Figs S3 and S4. A similar result was obtained with larval muscle walls. In contrast to the mitochondria in control muscle cells, the highly convoluted mitochondria in *hipk*-overexpressing muscle cells displayed increased MitoTracker Red incorporation, showing a sustained membrane potential (Fig. S5).

Tetramethylrhodamine methyl ester (TMRM) is another Δψ_m_-dependent, red fluorescent dye that accumulates in the matrix of active mitochondria (Scaduto and Grotyohann, 1999). Similar to the observations with mito-GFP (Fig. 1F-G), TMRM staining showed dot-like mitochondria in control cells and interconnected mitochondria in *hipk-*overexpressing salivary gland cells (Fig. 2J-K), again revealing the shift in mitochondrial morphology upon *hipk* overexpression. More importantly, cells with elevated Hipk showed significant TMRM sequestration compared with control cells (Fig. 2J,K,N). Mitochondrial membrane potential is established by pumping protons from the mitochondrial matrix to the intermembrane space. This can be achieved either by Complexes I/III/IV of the electron transport chain (ETC) in healthy functional mitochondria (Fig. 2A) or by the reversal of Complex V (ATP synthase) function (which occurs when the normal operation of the ETC is compromised) (Fig. 2A′) (Zorova et al., 2018). To identify the cause of membrane hyperpolarization in *hipk-*overexpressing cells, we used RNAi to knock down individually *pdsw* (also known as *ND-pdsw*) and *ATPsynβ*, which encode a Complex I subunit and a Complex V subunit, respectively (Fig. 2A). If Δψ_m_ is sustained by ETC Complexes I/III/IV, *pdsw* knockdown would suppress TMRM accumulation and *ATPsynβ* knockdown would maintain TMRM accumulation. By contrast, if Δψ_m_ is sustained by the reversal of Complex V function, *pdsw* knockdown would have little effect on TMRM staining whereas *ATPsynβ* knockdown would reduce the staining. We found that RNAi lines targeting *pdsw* (*pdsw-RNAi*), but not *ATPsynβ-RNAi*, markedly suppressed TMRM accumulation in *hipk-*overexpressing cells (Fig. 2L-N), inferring that Complex I is primarily required for sustaining the membrane potential. Additionally, the mitochondria in *hipk* and *pdsw-RNAi* co-expressing cells remained abundant (Fig. S2J), suggesting that the suppression of membrane hyperpolarization by *pdsw-RNAi* is not a consequence of loss of mitochondrial mass. Thus, we favor the model whereby Complexes I/III/IV in the ETC sustain the membrane potential in cells with elevated Hipk over the model in which the respiratory chain is compromised and Complex V functions in reverse to sustain the potential.

Together, by examining various aspects of mitochondrial metabolism (dynamics, abundance and energetics), we demonstrate that Hipk tumor-like cells are enriched with hyperfused and hyperpolarized mitochondria. In other words, we have established elevated Hipk as an *in vivo* fly tumor model characterized by a distinct mitochondrial profile, which may be useful in dissecting how mitochondrial metabolism is linked to tumor-like growth.

### Hipk tumor-like cells acquire the distinct mitochondrial profile through Myc and Pfk2

**Fig. 3.**
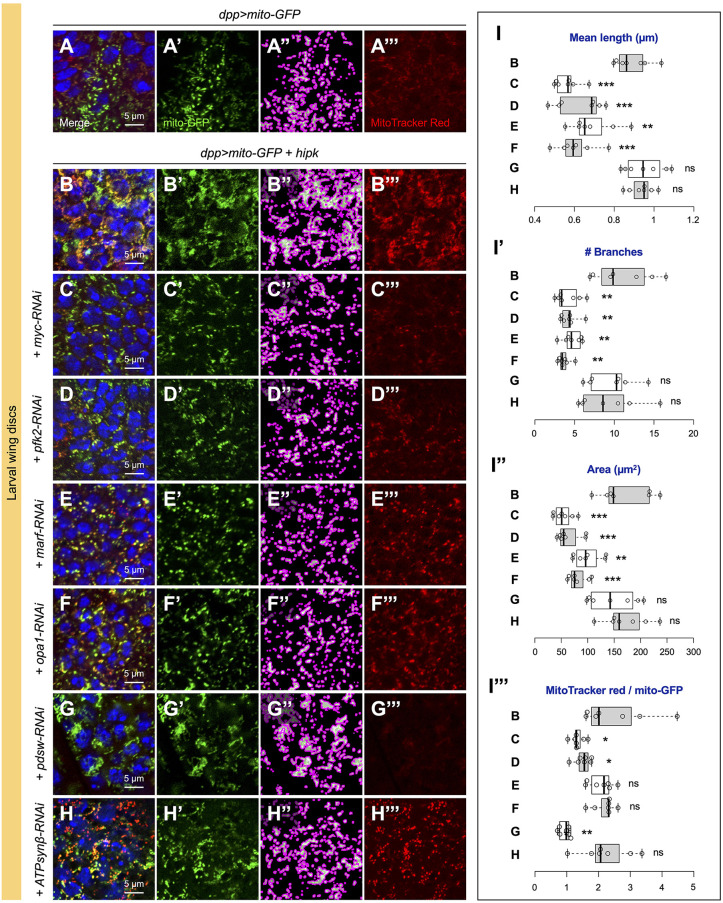
**Functional characterization of the altered mitochondrial profile in the *hipk*-overexpressing cells.** (A-H) Mitochondria (marked by mito-GFP in green) in DP cells of a control wing disc (*dpp>mito-GFP*) (A), wing discs overexpressing *hipk* alone (*dpp>mito-GFP+hipk*) (B) or with *myc* knockdown (*dpp>mito-GFP+hipk+myc-RNAi*) (C), *pfk2* knockdown (*dpp>mito-GFP+hipk+pfk2-RNAi*) (D), *marf* knockdown (*dpp>mito-GFP+hipk+marf-RNAi*) (E), *opa1* knockdown (*dpp>mito-GFP+hipk+opa1-RNAi*) (F), *pdsw* knockdown (*dpp>mito-GFP+hipk+pdsw-RNAi*) (G) or *ATPsynβ* knockdown (*dpp>mito-GFP+hipk+ATPsynβ-RNAi*) (H). Images in A′-H′ were processed by MiNA with mitochondrial outline shown in magenta and skeleton in green (A″-H″). Wing discs were stained with MitoTracker Red (red) (A‴-H‴). (I-I‴) Box and whisker plots showing the mean mitochondrial length (I), the number of branches (I′), the mitochondrial area (I″) and the ratio of MitoTracker Red to mito-GFP signal intensities (I‴) in wing discs of the indicated genotypes. Letters B-H refer to the genotypes shown in panels B-H. DNA (blue) was stained by DAPI. *P*-values were calculated using unpaired two-tailed *t-*test: *P<0.05, **P<0.01, ***P<0.001; ns, not significant.

Earlier, we showed that Hipk tumor-like cells manifested Myc-driven Warburg effect (aerobic glycolysis)-like metabolic changes, including increased glucose uptake and transcriptional activation of glycolytic genes (e.g. encoding phosphofructokinase-2; *pfk2*, also known as *pfrx*) (Wong et al., 2019). To find out whether the mitochondrial changes in Hipk tumor-like cells are linked to upregulation of Myc or Pfk2, we used RNAi to knock down Myc and Pfk2 individually in *hipk-*overexpressing wing discs and focused on transgene-expressing DP cells on the basal side of wing discs. In *hipk*-overexpressing wing discs, the tumor-like cells were enriched with fused mitochondria and intense MitoTracker Red staining (compare Fig. 3B with control in Fig. 3A). When *myc* or *pfk2* was knocked down (knockdown of which reduces glycolytic flux) in *hipk-*overexpressing cells, most mitochondria appeared punctate and MitoTracker Red staining was diminished (Fig. 3C,D). Analyses using the MiNA toolset show that knockdown of *myc* or *pfk2* markedly reduced mitochondrial length, branching, mass and membrane potential in *hipk-*overexpressing cells (Fig. 3I-I‴). To gather more solid evidence of the suppression effect, we examined salivary gland cells and comparable results were obtained (Fig. S6A-C). Thus, our data suggest that the changes in mitochondrial morphology, abundance and energetics in Hipk tumor-like cells require Myc and Pfk2.

**Fig. 4.**
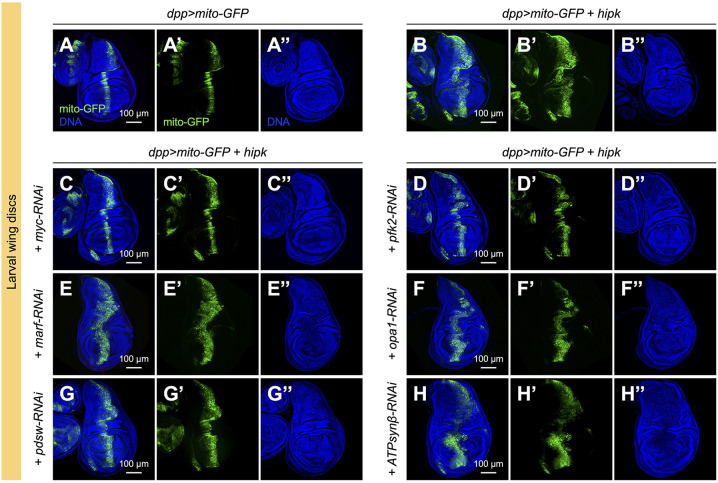
**Targeted inhibition of mitochondrial energetics suppresses Hipk-induced** **tumor-like growth.** (A-H) Whole tissue morphologies of a control wing disc (*dpp>mito-GFP*) (A), wing discs overexpressing *hipk* alone (*dpp>mito-GFP+hipk*) (B) or with *myc* knockdown (*dpp>mito-GFP+hipk+myc-RNAi*) (C), *pfk2* knockdown (*dpp>mito-GFP+hipk+pfk2-RNAi*) (D), *marf* knockdown (*dpp>mito-GFP+hipk+marf-RNAi*) (E), *opa1* knockdown (*dpp>mito-GFP+hipk+opa1-RNAi*) (F), *pdsw* knockdown (*dpp>mito-GFP+hipk+pdsw-RNAi*) (G) or *ATPsynβ* knockdown (*dpp>mito-GFP+hipk+ATPsynβ-RNAi*) (H). The transgene-expressing Dpp domain of each wing disc was marked by mito-GFP (green) (A′-H′). DNA (blue) was stained by DAPI (A″-H″).

When *hipk* was overexpressed in imaginal discs, the proliferation of tumor cells was accompanied by the formation of abnormal folds, as revealed by low magnification views of DNA staining (compare Fig. 4B with the control in Fig. 4A). Using tissue morphology as a functional readout for Hipk tumorigenic-like activity, we found that knockdown of *myc* or *pfk2* significantly suppressed the distortions in tissue morphology caused by elevated Hipk (Fig. 4C,D). This result is consistent with our earlier findings showing that Myc and Pfk2 are both required for Hipk-mediated tumor-like growth (Wong et al., 2019).

### Mitochondrial fusion is dispensable for Hipk-induced tumor-like growth

Next, we asked whether the shift in mitochondrial morphology plays a functional role during the process of Hipk-mediated tumor-like growth. To address this question, we genetically inhibited the fusion regulators Marf or Opa1 using RNAi to block mitochondrial fusion. When *marf* or *opa1* was knocked down in Hipk tumor-like cells, most mitochondria appeared dot-like (Fig. 3E,F,I-I″). Intriguingly, some mitochondria still sequestered relatively high levels of MitoTracker Red (Fig. 3E,F), and the ratios of MitoTracker Red to mito-GFP indicated that these fragmented mitochondria remained hyperpolarized even though fusion was inhibited (Fig. 3I‴). Similar results were obtained in salivary gland cells (Fig. S6D,E). These data hint at a model in which elevated Hipk alters mitochondrial morphology and membrane potential through separate mechanisms. Notably, when mitochondrial fusion was blocked, the disc morphologies remained distorted (Fig. 4E,F), suggesting that mitochondrial fusion may not be a key driver for Hipk-mediated tumor-like growth.

### Targeted inhibition of mitochondrial energetics suppresses Hipk-induced tumor-like growth

Using RNAi targeting Complex I and V subunits of the ETC, we next asked whether altered mitochondrial energetics are required for Hipk-mediated tumor-like growth. Genetic inhibition of Complex I by *pdsw-RNAi* resulted in diminished MitoTracker Red staining (Fig. 3G‴,I‴). Intriguingly, as revealed by mito-GFP, the knockdown did not suppress the mitochondrial fusion phenotype caused by elevated Hipk (Fig. 3G-G″). Thus, our data again imply that elevated Hipk impacts mitochondrial morphology and membrane potential independently. Most importantly, *pdsw-RNAi* restored the tissue morphology to wild type-like (Fig. 4G). This result implies that Complex I-dependent membrane hyperpolarization plays a crucial role in driving Hipk-mediated tumor-like growth.

We also tested the effects of RNAi targeting Complex V in wing discs. When *ATPsynβ-RNAi* was co-expressed in Hipk tumor-like cells to inhibit Complex V, the fusion phenotype seemed to be suppressed, because the mitochondria appeared less network-like (Fig. 3H). However, no statistically significant changes in mitochondrial length and branching were detected (Fig. 3I), possibly because the morphological change was too subtle or too heterogenous. In salivary gland cells, *ATPsynβ-RNAi* led to the formation of individual mitochondrial particles in the *hipk-*overexpression background (Fig. S6G). Notably, MitoTracker Red accumulation remained robust when *ATPsynβ* was knocked down (Fig. 3H‴,I‴; Fig. S6G), corroborating the TMRM results (Fig. 2M,N). Furthermore, *ATPsynβ* knockdown did not suppress Hipk-mediated tumor-like growth, as the disc morphology remained severely distorted (Fig. 4H). Thus, we propose that targeted inhibition of mitochondrial energetics (i.e. Pdsw inhibition) to reduce the membrane potential is required in order to suppress Hipk-mediated tumor-like growth.

### Complex subunit-dependent effects on ROS production in Hipk tumor-like cells

In an attempt to resolve why *pdsw-RNAi*, but not by *ATPsynβ-RNAi*, can suppress Hipk-mediated tumor-like growth, we assessed the levels of reactive oxygen species (ROS) in the wing discs because we might have introduced unintended mitochondrial dysfunction to the tumor-like cells in these RNAi experiments.

**Fig. 5.**
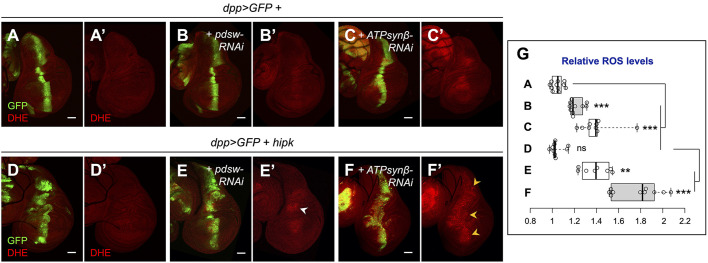
**Complex subunit-dependent effects on ROS production.** (A-F) DHE staining to reveal ROS buildup (red) of wing discs of the indicated genotypes: *dpp>GFP* (A), *dpp>GFP+pdsw-RNAi* (B), *dpp>GFP+ATPsynβ-RNAi* (C), *dpp>GFP+hipk* (D), *dpp>GFP+hipk+pdsw-RNAi* (E) and *dpp>GFP+hipk+ ATPsynβ-RNAi* (F). GFP (green) marks the transgene-expressing cells. (G) Box and whisker plot showing the relative ROS levels of the transgene-expressing cells of the indicated genotypes. Letters A-F refer to the genotypes shown in panels A-F. *P*-values were calculated using unpaired two-tailed *t-*test: **P<0.01, ***P<0.001; ns, not significant. Scale bars: 50 μm.

To measure ROS levels, we stained the wing discs with dihydroethidium (DHE), which is a fluorogenic probe for superoxide (O_2_^– •^). DHE staining revealed the basal ROS levels in control wing discs (Fig. 5A). In *hipk-*overexpressing cells, despite the tumorous growth, the ROS levels were comparable to the basal levels in control discs (compare Fig. 5D with Fig. 5A; Fig. 5G). Disruption of mitochondrial complex function is usually associated with massive generation of ROS (Owusu-Ansah and Banerjee, 2009). Thus, the lack of ROS buildup in Hipk tumor cells suggests that the tumor mitochondria remain functional, rather than damaged.

When *pdsw* was knocked down in the control background (without *hipk* overexpression), we observed 10-20% increase in ROS levels in the knockdown cells relative to the neighboring control cells (Fig. 5B,G), consistent with a previous study showing elevated ROS production upon Complex I inhibition (Perez-Gomez et al., 2020). Knockdown of *ATPsynβ* increased the relative ROS levels by 30-40% (Fig. 5C,G). This confirms the model that mitochondrial dysfunction generally brings about ROS production. When co-expressed in Hipk tumor-like cells, *pdsw-RNAi* induced mild ROS in or close to the hinge region of the wing discs (30-40% increase) (Fig. 5E′, white arrowhead; Fig. 5G), whereas *ATPsynβ-RNAi* led to robust ROS production (∼70% increase) across nearly the entire *hipk-*overexpressing domain (Fig. 5F′, orange arrowheads; Fig. 5G). Our results reflect a differential effect on ROS production by different RNAi lines targeting mitochondrial energetics.

### Generic inhibition of mitochondrial energetics suppresses Hipk-induced tumor-like growth more effectively when ROS scavenging enzymes are overproduced

**Fig. 6.**
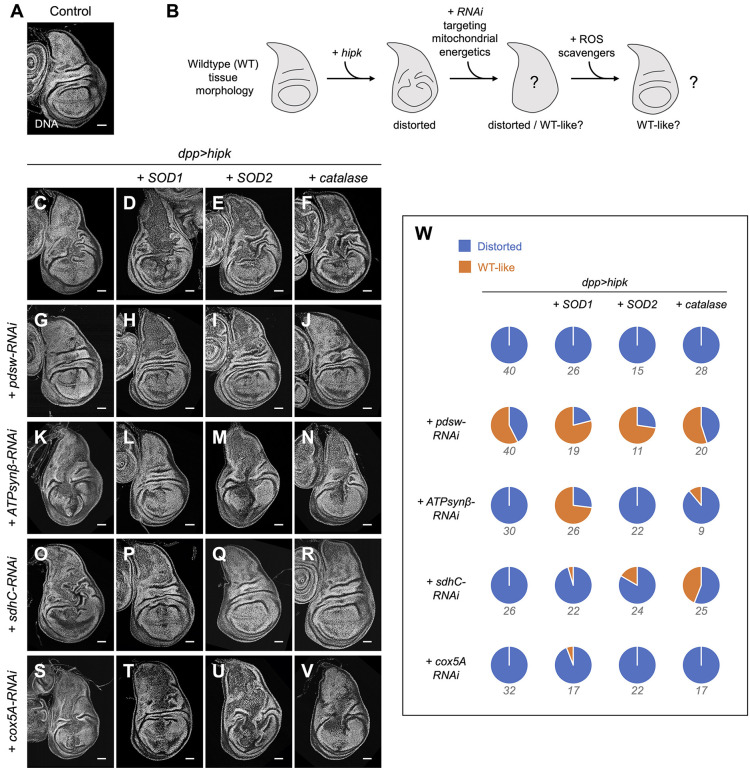
**Generic inhibition of mitochondrial energetics suppresses Hipk-induced tumor-like growth more effectively when ROS scavenging enzymes are overproduced.** (A) The normal tissue morphology of a control wing disc is shown by DNA staining. (B) The use of a morphology assay to identify the conditions under which the tumor-like growth (as revealed by the distortions in tissue morphology) can be suppressed. (C-V) Disc morphologies of the indicated genotypes are shown by DNA staining: (C) *dpp>hipk*, (D) *dpp>hipk+SOD1*, (E) *dpp>hipk+SOD2*, (F) *dpp>hipk+catalase*, (G) *dpp>hipk+pdsw-RNAi*, (H) *dpp>hipk+SOD1+pdsw-RNAi*, (I) *dpp>hipk+SOD2+pdsw-RNAi*, (J) *dpp>hipk+catalase+pdsw-RNAi*, (K) *dpp>hipk+ATPsynβ-RNAi*, (L) *dpp>hipk+SOD1+ ATPsynβ-RNAi*, (M) *dpp>hipk+SOD2+ATPsynβ-RNAi*, (N) *dpp>hipk+catalase+ATPsynβ-RNAi*, (O) *dpp>hipk+sdhC-RNAi*, (P) *dpp>hipk+SOD1+sdhC-RNAi*, (Q) *dpp>hipk+SOD2+sdhC-RNAi*, (R) *dpp>hipk+catalase+sdhC-RNAi*, (S) *dpp>hipk+cox5A-RNAi*, (T) *dpp>hipk+SOD1+cox5A-RNAi*, (U) *dpp>hipk+SOD2+cox5A-RNAi* and (V) *dpp>hipk+catalase+cox5A-RNAi.* (W) Pie charts showing quantification of the tissue morphology phenotypes of the indicated genotypes. The number of discs examined per genotype is shown below the corresponding pie chart. Scale bars: 50 μm.

Based on the ROS experiments described above, we hypothesized that the high ROS generated as a consequence of *ATPsynβ-RNAi* expression explains why inhibition of mitochondrial energetics by this knockdown fails to suppress Hipk-mediated tumor-like growth. To test our hypothesis, we used the tissue morphology assay (used in Fig. 4) to test whether RNAi targeting mitochondrial energetics can restore the distorted morphology back to wild type-like and suppress Hipk-mediated tumor-like growth (Fig. 6B). Furthermore, we tested whether RNAi can prevent the tumor-like growth in the presence of overexpressed ROS scavengers, which include superoxide dismutase 1 (SOD1), SOD2 and catalase. The cytosolic SOD1 and the mitochondrial SOD2 convert superoxide radical (O_2_^– •^) into less reactive H_2_O_2_, whereas catalase catalyzes the conversion of H_2_O_2_ into water (Wang et al., 2018).

We noticed that none of the ROS scavengers tested could suppress the distortions in tissue morphology caused by elevated Hipk (Fig. 6C-F,W). This result was expected because we detected no overproduction of ROS in Hipk tumor-like cells (Fig. 5D), indicating that the tumor-like growth is not driven by ROS when *hipk* is overexpressed on its own. When *pdsw-RNAi* was expressed to inhibit Complex I in the tumor-like cells, the distortion phenotype was largely suppressed as over half of the wing discs showed a wild type-like morphology, regardless of the expression levels of ROS scavengers (Fig. 6G-J,W). As we showed previously, inhibition of Complex V by *ATPsynβ-RNAi* failed to rescue Hipk tumor growth (Fig. 6K). However, this *ATPsynβ-RNAi* was able to suppress the distortion phenotype in some wing discs under the conditions of elevated SOD1 and catalase, but not when SOD2 was elevated (Fig. 6L-N,W). We also tested two additional RNAi lines targeting Complex II (*sdhC-RNAi*) and Complex IV (*cox5A-RNAi*). Without the overexpression of ROS scavengers, neither suppressed the Hipk-induced distortion phenotype (Fig. 6O,S,W). When ROS scavengers were overexpressed, expression of *sdhC-RNAi* blocked Hipk-induced tumor-like growth to a certain extent (Fig. 6P-R,W). By contrast, *cox5A-RNAi* expression showed a minor suppression effect only in the presence of excess SOD1 (Fig. 6T-W). Hence, our data suggest that in order to suppress Hipk-induced tumor-like growth by inhibition of mitochondrial energetics, the ROS levels produced in the tumor-like cells need to be kept low.

### *ATPsynβ* knockdown potentiates JNK activation in Hipk tumor-like cells

**Fig. 7.**
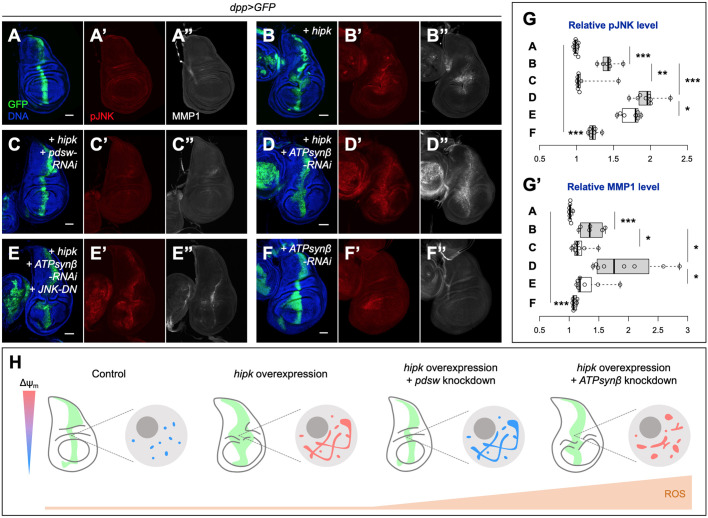
**ATPsynβ knockdown potentiates JNK activation in Hipk tumor-like cells.** (A-F) pJNK (red in A′-F′) and MMP1 (gray in A″-F″) staining in a control wing disc (*dpp>GFP*) (A), wing discs overexpressing *hipk* alone (*dpp>GFP+hipk*) (B) or with *pdsw* knockdown (*dpp>GFP+hipk+pdsw-RNAi*) (C), *ATPsynβ* knockdown (*dpp>GFP+hipk+ATPsynβ-RNAi*) (D) or *ATPsynβ* knockdown plus *JNK-DN* overexpression (*dpp>GFP+hipk+ATPsynβ-RNAi+JNK-DN*) (E), and in a *ATPsynβ-RNAi* wing disc (*dpp>GFP+ATPsynβ-RNAi*) (F). The transgene-expressing Dpp domain of each disc was labeled by GFP (green). DNA was stained with DAPI (blue). (G) A box and whisker plot showing the relative pJNK (G) and MMP1 (G′) levels of the transgene-expressing cells of the indicated genotypes. Letters A-F refer to the genotypes shown in panels A-F. (H) Diagram showing *hipk* overexpression as an *in vivo* tumor model featuring the accumulation of hyperfused and hyperpolarized mitochondria. Inhibitions of the respiratory Complex subunits Pdsw and ATPsynβ have varying effects on mitochondrial morphology, energetics, ROS production and the tumor-like growth. Knockdown of *pdsw* lowers Δψ_m_, induces moderate ROS and blocks the tumor-like growth. By contrast, *ATPsynβ* knockdown causes mitochondrial fragmentation, sustains Δψ_m_, induces high ROS and permits the tumor-like growth with potentiation of JNK activity. Scale bars: 50 μm.

Finally, we examined the effects of high ROS production on Hipk-induced tumor-like growth as a model of what might occur during advanced tumorigenesis. In line with our previous studies (Blaquiere et al., 2018), compared with control discs (Fig. 7A), elevated Hipk on its own triggered activation of the JNK signaling pathway, as revealed by phosphorylated JNK (pJNK) staining (Fig. 7B′,G), as well as induction of MMP1 (*mmp1* is a target gene of JNK) (Fig. 7B″,G′). However, JNK activation in *hipk-*overexpressing discs was limited to the hinge region (Fig. 7B). When *pdsw-RNAi* was co-expressed in *hipk-*overexpressing cells, tumor-like growth, JNK phosphorylation and MMP1 induction were significantly suppressed (Fig. 7C,G). By contrast, *ATPsynβ* knockdown in Hipk tumor-like cells not only generated high ROS (Fig. 5F), but also potentiated JNK activation and MMP1 upregulation across the entire Hipk-expression domain (Fig. 7D,G), and the effects seemed both additive and synergistic because *ATPsynβ-RNAi* on its own induced mild JNK phosphorylation (Fig. 7F). Genetic inhibition of JNK by overexpression of a dominant negative form of JNK (JNK-DN) largely suppressed MMP1 upregulation in the *hipk* and *ATPsynβ-RNAi* co-expressing background, although JNK-DN remained highly phosphorylated (Fig. 7E,G). MMP1 is responsible for degrading basement membrane proteins and facilitating tumor invasion (Page-McCaw et al., 2007). Thus, our data suggest that when high ROS is induced in Hipk tumor-like cells as a result of inhibition of mitochondrial energetics, the high ROS can potentiate JNK activation and downstream MMP1 induction, which probably triggers tumor invasion and exacerbates tumor progression.

In summary, we propose a model whereby, in Hipk tumor-like cells enriched with hyperpolarized mitochondria, targeting the mitochondrial energetics can reduce tumor-like growth if ROS levels remain at a moderate level (Fig. 7H). Otherwise, high ROS induces aberrant cell signaling such as JNK activation and sustains tumor-like growth. In the latter scenario, simultaneous inhibition of mitochondrial energetics and scavenging of ROS are required to prevent growth.

## DISCUSSION

### Elevated Hipk as an *in vivo* tumor model with accumulation of hyperfused and hyperpolarized mitochondria

Mitochondrial proteins involved in the ETC and oxidative phosphorylation are required for normal growth and development of animals; for example, the larval wing and eye discs in *Drosophila* (Perez-Gomez et al., 2020; Pletcher et al., 2019)*.* Mitochondrial dysfunction has been implicated in numerous fly tumor models (Van Den Ameele and Brand, 2019; Ji et al., 2019; Katheder et al., 2017; Lee et al., 2013; Wang et al., 2016). Here, we present elevation of Hipk as a tumor model with a distinct mitochondrial profile. Characterization of the mitochondrial metabolism in Hipk tumor-like cells reveals a switch in morphology from fission to fusion (Fig. 1), an increase in the mitochondrial mass (Fig. 1), membrane hyperpolarization (Fig. 2) and a lack of ROS buildup (Fig. 5), suggesting that the mitochondria in Hipk tumor-like cells are functional rather than damaged. The Hipk-induced mitochondrial changes in the wing disc cells are not cell-type specific, as comparable changes were observed in salivary gland cells and muscle cells (Figs 1,2; Figs S2,S5,S6).

Notably, knockdown of *Myc* or *Pfk2* in the tumor cells suppressed the mitochondrial changes and tumor-like growth in *hipk-*overexpressing discs (Fig. 3A-D, Fig. 4A-D). Myc, a well-known oncoprotein, plays conserved roles in cell growth, aerobic glycolysis and mitochondrial biogenesis (Li et al., 2005; Stine et al., 2015). In *Drosophila*, several genes encoding mitochondrial ribosomal proteins have been found to be Myc responsive (Orian et al., 2003). The fusion regulator genes, *marf* and *opa1*, together with *Myc*, are target genes of Yki (Nagaraj et al., 2012; Neto-Silva et al., 2010), and Hipk is a positive regulator of Yki (Chen and Verheyen, 2012; Poon et al., 2012). Thus, it is conceivable that elevated Hipk promotes mitochondrial biogenesis and fusion through the combined actions of Myc and Yki. In addition, because Myc and aerobic glycolysis reciprocally stimulate each other (Wong et al., 2019), aerobic glycolysis (stimulated by Pfk2) probably maintains the changes in mitochondrial profile through Myc. Thus, Hipk-induced tumor-like growth and the associated metabolic changes seem to be acquired through integrated cell signaling and metabolic pathways.

### Regulation of mitochondrial fusion and energetics by separate mechanisms

Mitochondrial morphology in general reflects the mitochondrial and cellular bioenergetic states, as fused mitochondria are more prevalent in respiratory active cells (Westermann, 2012). Interestingly, we noted that blocking mitochondrial fusion did not significantly suppress the membrane hyperpolarization in Hipk tumor-like cells (Fig. 3E,F). Furthermore, *pdsw* knockdown-mediated reduction in membrane potential did not reverse the fusion phenotype (Fig. 3G). More importantly, *pdsw* knockdown abrogated Hipk-induced tumor-like growth, whereas blocking mitochondrial fusion had negligible effects (Fig. 4). This implies that mitochondrial energetics play a more predominant role than mitochondrial morphology in driving the tumor-like growth. Our data also point to a model in which mitochondrial morphology and energetics are governed separately and can be uncoupled from each other by genetic manipulation. This model is in line with recent findings showing that neuronal health depends on functional mitochondria, regardless of their shape (Trevisan et al., 2018). Nevertheless, our observation that the highly fused mitochondria in Hipk tumor-like cells were hyperpolarized illustrates that the changes in the mitochondrial profile are achieved in a coordinated manner even though separate mechanisms are involved.

### ROS levels underlie the paradoxical effects of mitochondrial inhibition on tumor growth

Upon perturbation of mitochondrial energetics, we observed opposing outcomes on the tumor-like growth. Knockdown of *pdsw*, but not *ATPsynβ*, *sdhC* or *cox5A*, abrogated Hipk-induced tumor-like growth (Figs 4,6). Similar confounding results have been reported previously. For example, loss of the mammalian *NDUFB10* (*pdsw* in *Drosophila*) reduces the growth of K-RAS tumors (Martin et al., 2017), whereas low expression of *NDUFS1* (which encodes another Complex I subunit) is correlated with poor prognosis and metastasis in non-small cell lung cancer patients (Su et al., 2016). Intriguingly, we observed that when mitochondrial energetics are inhibited by knockdown of different complex subunits, the amounts of ROS produced varied. Expression of *pdsw-RNAi* and *ATPsynβ-RNAi* led to mild and high ROS, respectively (Fig. 5). The differential effect of the knockdowns could presumably be determined by knockdown efficiency or the importance of the complex subunits in ROS production. Given that high ROS potentiates JNK-dependent MMP1 induction (Fig. 7), most of the RNAi lines tested (*ATPsynβ-RNAi*, *sdhC-RNAi* and *cox5A-RNAi*) need to be accompanied by simultaneous overexpression of ROS scavengers in order to reduce Hipk tumor growth (Fig. 6). Thus, our work implies that the ability of inhibition of mitochondrial energetics to suppress Hipk-induced tumor-like growth depends on whether the levels of ROS surpass a threshold to cause deleterious effects.

In summary, we establish elevated Hipk as a tumor model characterized by a Complex I-sustained membrane hyperpolarization, which is distinct from the previously described models with reduced mitochondrial activity. Using this Hipk tumor model, we demonstrate that targeted inhibition of mitochondrial activity is required to suppress Hipk-induced tumor-like growth, as the generic inhibition may paradoxically generate massive ROS production, which sustains tumor-like progression.

## MATERIALS AND METHODS

### *Drosophila* culture

Flies were raised on standard cornmeal-molasses food. Crosses were kept at 29°C unless otherwise indicated. Two Gal4 fly lines, (1) *dpp-Gal4/TM6B* and (2) *mef2-Gal4 III*, were used to induce transgene expression. (3) *UAS-HA-hipk-3M* (abbreviated as *UAS-hipk*) (Swarup and Verheyen, 2011) and (4) *dpp-Gal4 UAS-hipk* (Blaquiere et al., 2018) were used to generate the Hipk tumor model (abbreviated as *dpp>hipk*). (5) *UAS-GFP* (BL 5431) was used to mark the transgene-expressing cells. To visualize the mitochondrial morphology, (6) *UAS-mito-HA-GFP* (abbreviated as *UAS-mitoGFP*; BL 8442) was used. RNAi fly strains used included (7) *UAS-marf-RNAi* (BL 55189), (8) *UAS-opa1-RNAi* (BL 32358), (9) *USA-ND-pdsw-RNAi* (BL 29592), (10) *USA-ATPsynβ-RNAi* (BL 28056), (11) *UAS-JNK-DN* (also known as *UAS-bsk-DN*, BL 6409), (12) *UAS-dMyc-RNAi* (BL 25783), (13) *UAS-pfk2-RNAi* (BL 35380), (14) *UAS-sdhC-RNAi* (BL 53281) and (15) *UAS-cox5A-RNAi* (BL 27548). Strains with BL stock number were obtained from Bloomington *Drosophila* Stock Center (Bloomington, IN, USA).

### Immunofluorescence staining

Larval imaginal discs were dissected in PBS and fixed in 4% paraformaldehyde (PFA) for 15 min at room temperature. Samples were washed with PBS containing 0.1% Triton X-100 (PBST). After blocking with 5% BSA in PBST for 1 h at room temperature, samples were incubated with primary antibodies overnight at 4°C. The following primary antibodies were used: rabbit anti-pJNK (pTPpY) (1:500; Promega V7931) and mouse anti-Mmp1 (1:100; 3A6B4, 3B8D12, 5H7B11; DSHB; deposited by G. M. Rubin, HHMI/Janelia Farm Research). After washing with PBST, samples were incubated with Cy3- and/or Alexa Fluor 647-conjugated secondary antibodies (1:500; Jackson ImmunoResearch Laboratories), DAPI (final concentration: 0.2 μg per ml; Invitrogen D1306) for 2 h at room temperature. Samples were mounted in 70% glycerol in PBS after washing. Images were taken on a Nikon Air laser-scanning confocal microscope or a Zeiss LSM880 with Airyscan confocal microscope and processed using ImageJ.

### Quantification of mitochondrial morphology

The quantification was done using an ImageJ macro tool known as Mitochondrial Network Analysis (MiNA). In Airyscan images of salivary gland cells (∼67×67 μm^2^, 2024×2024 pixels^2^), areas (∼13×13 μm^2^, 400×400 pixels^2^ for each area) were selected and subject to the MiNA analyses using the following settings: CLAHE: block size 127, histogram bins 5, maximum slope 5; Median Filter: radius 2; Unsharp Mask: radius 2, mask strength 0.60; Tophat was not applied for processing. Mitochondrial morphology was quantified using the following four parameters: number of individuals (structure with no junctions), numbers of networks (structure with at least one junction), mean length of network branches (mean branch length) and mean number of branches per network (mean network size). Data collected from a total of 15 cells per genotype were pooled together for analyses and generating boxplots. For confocal images of the mitochondria in wing disc epithelial cells, the size of each area selected was ∼26.5×26.5 μm^2^ (250×250 pixels^2^).

### MitoTracker Red staining

Tissues of interest from third instar larvae were dissected in PBS, followed by incubation with MitoTracker Red CMXRos (Life Technologies, M7512) at 200 nM for 45 min in the dark at room temperature. After rinsing with PBS, tissue samples were fixed and processed according to a standard immunofluorescence staining protocol.

### TMRM staining

Larval salivary glands were dissected in PBS, followed by incubation with tetramethylrhodamine methyl ester (TMRM) perchlorate (Cayman 21437) at 100 nM for 30 min in the dark at room temperature. After rinsing with PBS briefly, samples were immediately subjected to live cell imaging on a Nikon Air laser-scanning confocal microscope.

### ROS assay

The ROS assays were performed according to the previously described protocol for *in vivo* detection of ROS (Owusu-Ansah et al., 2008). Briefly, larval wing discs were dissected in PBS and incubated with 30 µM DHE (Cayman 12013) for 7 min in the dark at room temperature. After rinsing with PBS briefly, samples were fixed in 8% PFA and washed with PBS, each for 5 min before mounting. Images were taken within 2 days.

### Statistics

For data analyses, unpaired two-tailed Student *t*-tests were used to determine *P*-values using Microsoft Excel. Boxplots were generated using BoxPlotR with data points included (Spitzer et al., 2014). We used the Spear definition to show the maximum and minimum values by the upper and lower whiskers, respectively. The third quartile, median and the first quartile are shown in the box according to a standard boxplot.

## Supplementary Material

Supplementary information

Reviewer comments
