# Supplementary material for: Hyperpolarized mitochondria accumulate in Drosophila Hipk-overexpressing cells to drive tumor-like growth
Source: J Cell Sci. 2020 Dec 9;133(23):jcs250944. doi: 10.1242/jcs.250944 (PMC7746665; doi:10.1242/jcs.250944)
Supplement: Supplementary information [file joces-133-250944-s1.pdf]

Supplemental figures

Figure S1. Elevated Hipk induces cell invasion-like behaviors.

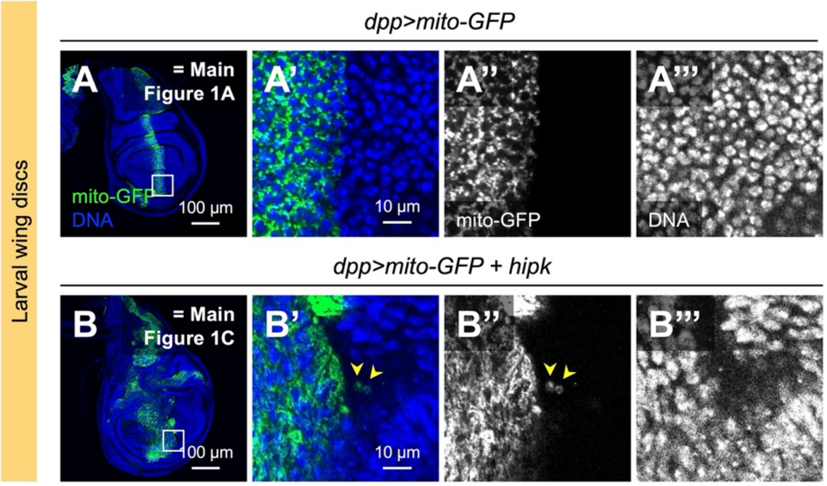

Control (*dpp > mito-GFP*) **(A)** and *hipk*-overexpressing (*dpp > mito-GFP + hipk*) **(B)** wing discs expressing mito-GFP (marking mitochondria in green; grey in **A''** and **B''**) under the control of *dpp-Gal4.* DNA was stained with DAPI (blue; grey in **A'''** and **B'''**) to reveal overall tissue morphology. Insets (solid line) in **A** and **B** are magnified in **A'-A'''** and **B'-B'''**, respectively. The *hipk*-overexpressing cells that display invasion-like phenotypes are marked by yellow arrowheads in **B'-B''**. Scale bars are shown as indicated.

Figure S2. Effects on mitochondrial morphology and mass by elevated Hipk and various knockdowns.

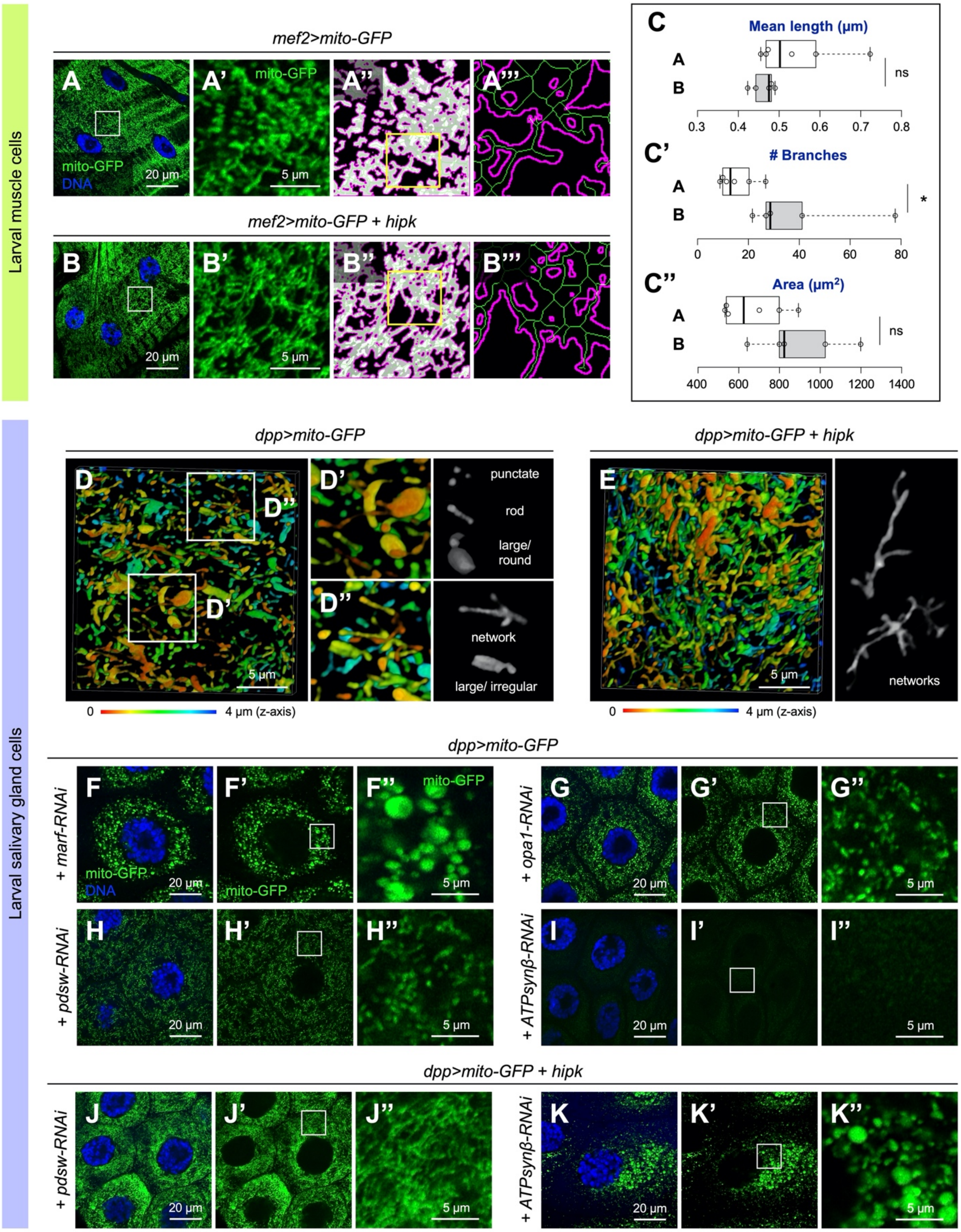

Journal of Cell Science • Supplementary information

(A-B) Control (*mef2 > mito-GFP*) **(A)** and *hipk*-overexpressing (*mef2 > mito-GFP + hipk*) **(B)** muscle cells expressing mito-GFP (marking mitochondria in green) under the control of *mef2-Gal4.* Insets (white) in **A** and **B** are magnified in **A'** and **B'**, respectively. Images **A'-B'** were processed by MiNA with mitochondrial outline shown in magenta and skeleton in green **(A''-B'', A'''-B''')**. Insets (yellow) in **A''** and **B''** are magnified in **A'''** and **B'''**, respectively.

(C) Box and whisker plots showing the mean mitochondrial length **(C)**, the number (#) of branches **(C')**, and the mitochondrial area **(C'')** in control (*dpp > mito-GFP*) and *hipk*-overexpressing (*dpp > mito-GFP + hipk*) larval muscle walls. Letters **A-B** refer to the genotypes shown in **Fig. S2A-B**.

(D-E) 3D images of mitochondria (marked by mito-GFP, color-coded for depth) in control (*dpp > mito-GFP*) **(D)** and *hipk*-overexpressing (*dpp > mito-GFP + hipk*) salivary gland cells **(E)**. Insets in **D** are magnified in **D'** and **D''**. Selected mitochondria of various shapes are highlighted and shown in grey.

(F-K) Airyscan images of mitochondria (marked by mito-GFP in green) in salivary gland cells of the indicated genotypes: **(F)** *dpp > mito-GFP + marf-RNAi*, **(G)** *dpp > mito-GFP + opa1-RNAi*, **(H)** *dpp > mito-GFP + pdsw-RNAi*, **(I)** *dpp > mito-GFP + ATPsynβ-RNAi*, **(J)** *dpp > mito-GFP + hipk + pdsw-RNAi* and **(K)** *dpp > mito-GFP + hipk + ATPsynβ-RNAi*. Insets in **F'-K'** are magnified in **F''-K''**.

DNA was stained with DAPI (blue). Scale bars are shown as indicated. *p* values calculated using unpaired two-tailed *t*-test are shown; * < 0.05, ** < 0.01, *** < 0.001.

Journal of Cell Science • Supplementary information

Figure S3. MitoTracker Red staining in a control wing disc.

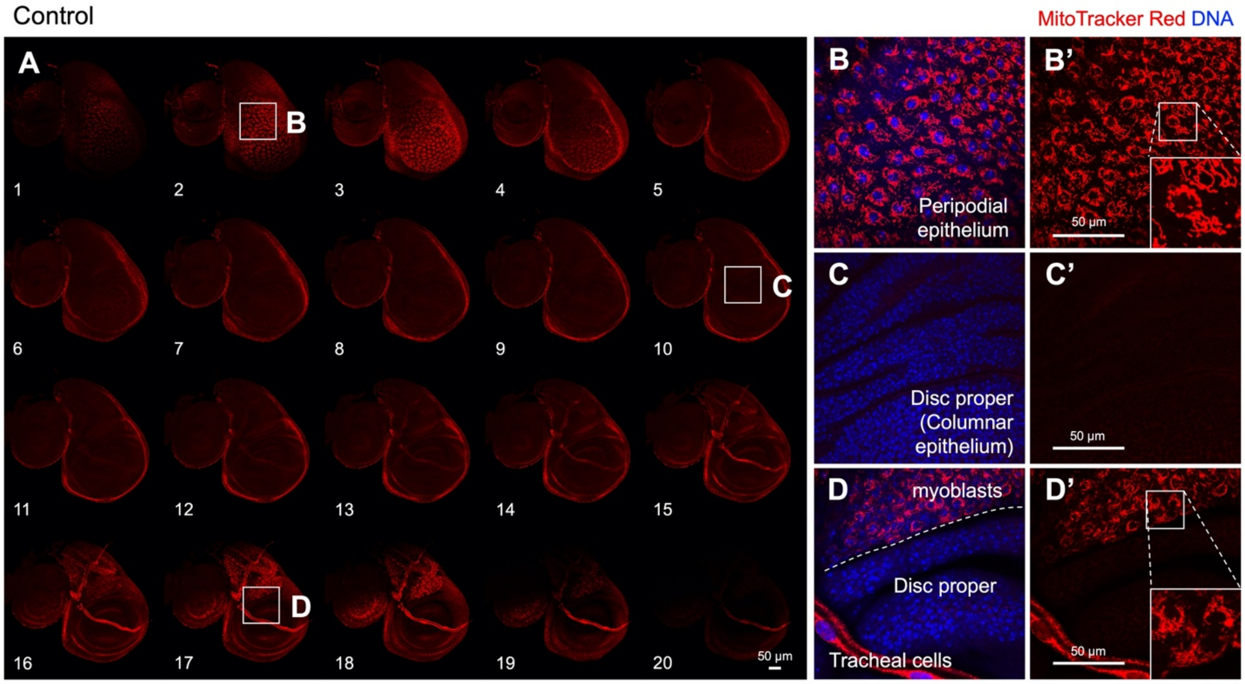

(A) Gallery view of a series of z-stack images showing Mitotracker Red staining (red) in a control wing disc from the apical side (starting from stack **1**) to the basal side. Insets (solid line) in stacks **2**, **10** and **17** are magnified in **B-D**. In **D**, a dashed line separates the myoblasts from the disc proper cells. DNA was stained with DAPI (blue). Scale bars, 50 µm.

Journal of Cell Science • Supplementary information

Figure S4. MitoTracker Red staining in a *hipk*-overexpressing wing disc.

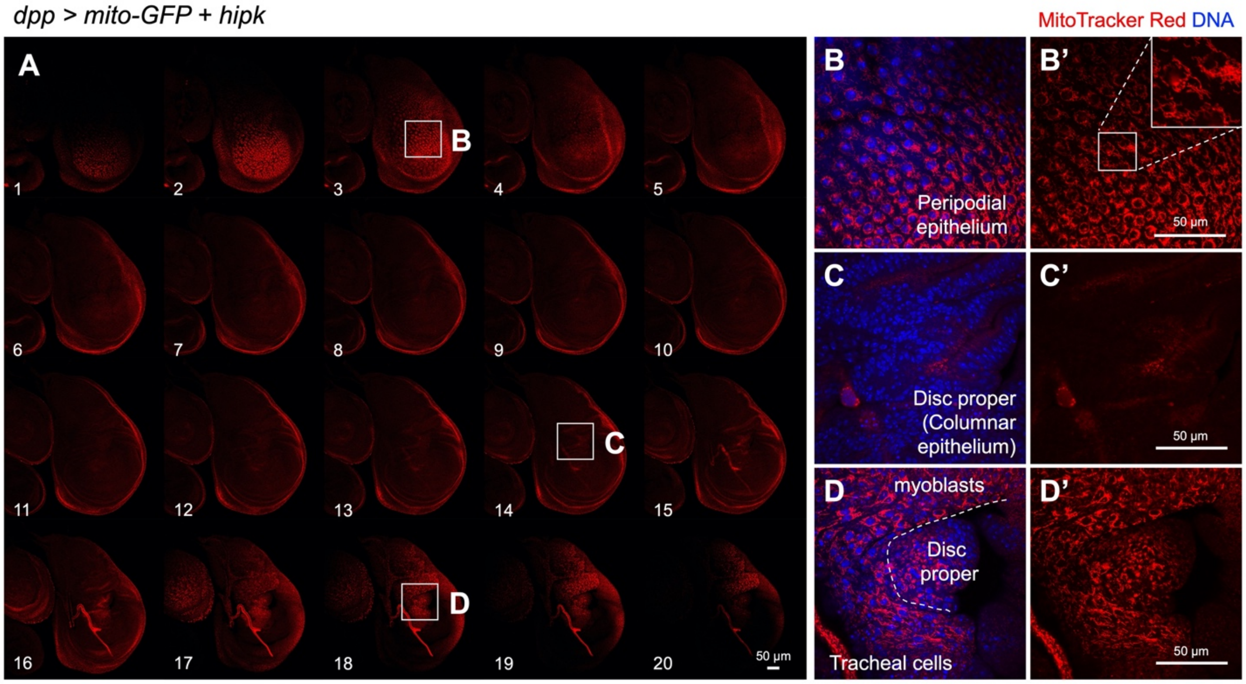

(A) Gallery view of a series of z-stack images showing Mitotracker Red staining (red) in a *hipk*-overexpressing wing disc from the apical side (starting from stack **1**) to the basal side. Insets (solid line) in stacks **3**, **14** and **18** are magnified in **B-D**. In **D**, a dashed line separates the myoblasts from the disc proper cells. We noticed that some myoblasts moved from the notum region into the hinge and pouch regions, possibly due to the tissue distortions caused by the tumor-like growth. Myoblasts are marked GFP-negative due to the absence of Gal4 expression driven by *dpp-Gal*. Also, myoblasts are larger in size and spindle-like in shape when compared with the squeezed, columnar disc proper cells. DNA was stained with DAPI (blue). Scale bars, 50 μm.

Journal of Cell Science • Supplementary information

Figure S5. Elevated Hipk promotes mitochondrial membrane hyperpolarization in larval muscle cells.

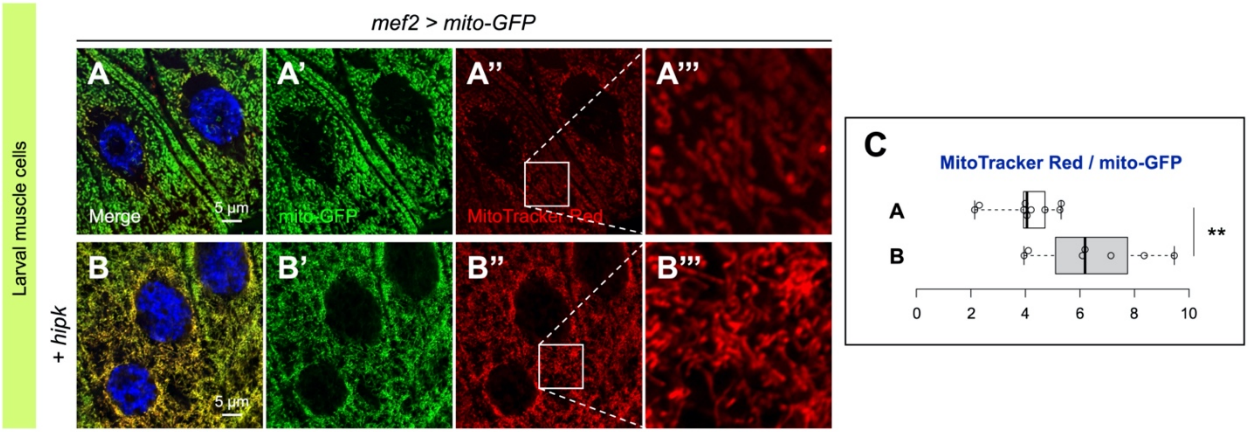

(A-B) Control (*mef2 > mito-GFP*) **(A)** and *hipk*-overexpressing (*mef2 > mito-GFP* + *hipk*) **(B)** larval muscle cells stained with MitoTracker Red (red). Mitochondria were marked by mito-GFP (green). DNA (blue) was stained with DAPI. Insets in **A''-B''** are magnified in **A'''-B'''**. Scale bars, 5 µm.

(C) A box and whisker plot showing the ratio of MitoTracker Red to mito-GFP signal intensities of control and *hipk*-overexpressing larval muscle cells. Letters **A-B** refer to the genotypes shown in **Fig. S5A-B**.

Journal of Cell Science • Supplementary information

Figure S6. Effects of various knockdowns on MitoTracker Red incorporation and mitochondrial morphology in *hipk*-overexpressing salivary gland cells.

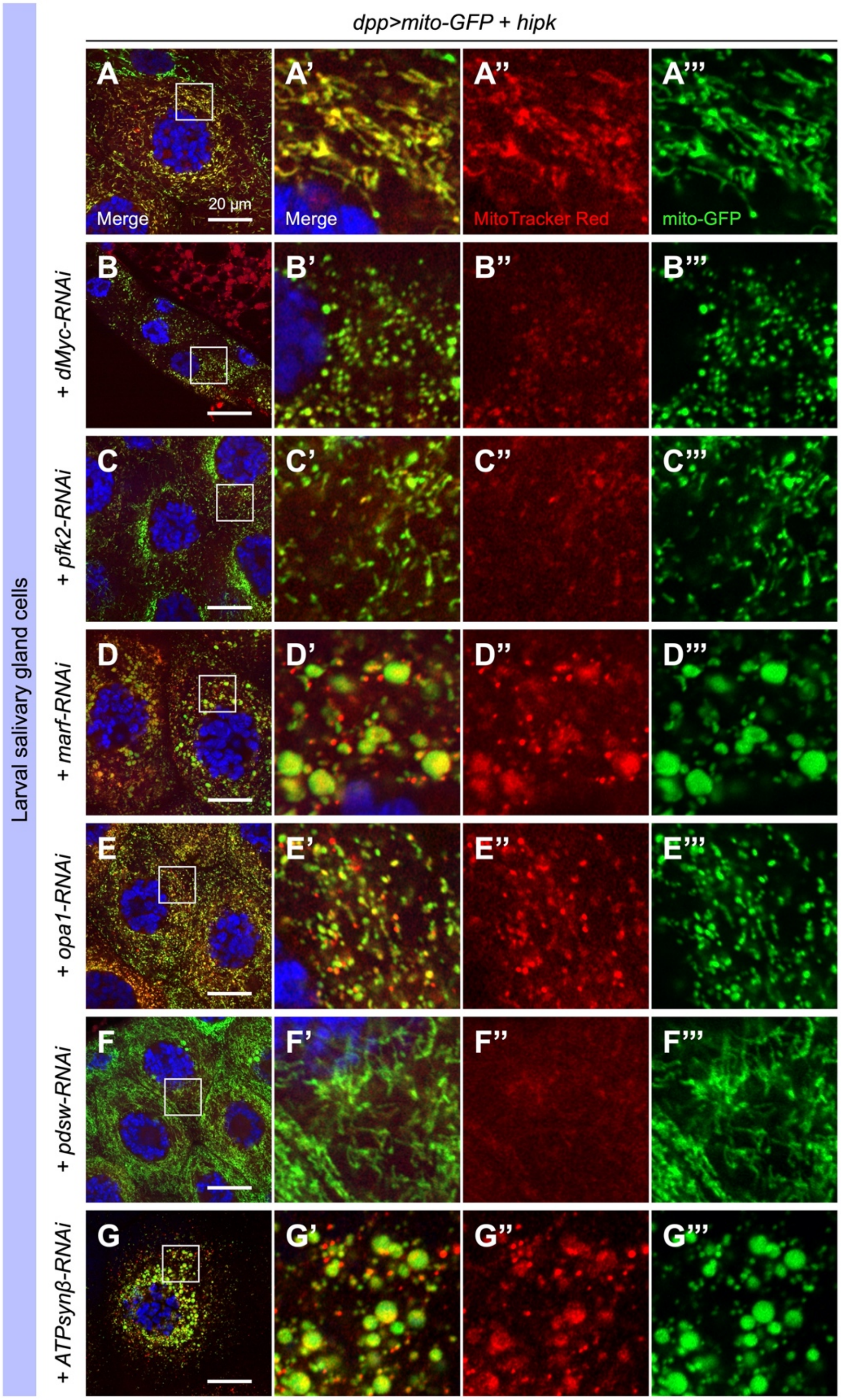

Mitochondria (marked by mito-GFP in green) and MitoTracker staining (red) in *hipk*-overexpressing salivary gland cells (*dpp* > *mito-GFP* + *hipk*) without **(A)** or with *dMyc* (or *Myc*)

Journal of Cell Science • Supplementary information

knockdown (*dpp > mito-GFP + hipk + dMyc-RNAi*) **(B)**, with *pfk2* knockdown (*dpp > mito-GFP + hipk + pfk2-RNAi*) **(C)**, with *marf* knockdown (*dpp > mito-GFP + hipk + marf-RNAi*) **(D)**, with *opa1* knockdown (*dpp > mito-GFP + hipk + opa1-RNAi*) **(E)**, with *pdsw* knockdown (*dpp > mito-GFP + hipk + pdsw-RNAi*) **(F)**, or with *ATPsynβ* knockdown (*dpp > mito-GFP + hipk + ATPsynβ-RNAi*) **(G)**. Insets in **A-G** are magnified in **(A'-A''')-(G'-G''')**. DNA (blue) was stained with DAPI. Scale bars, 20 μm.

Journal of Cell Science • Supplementary information

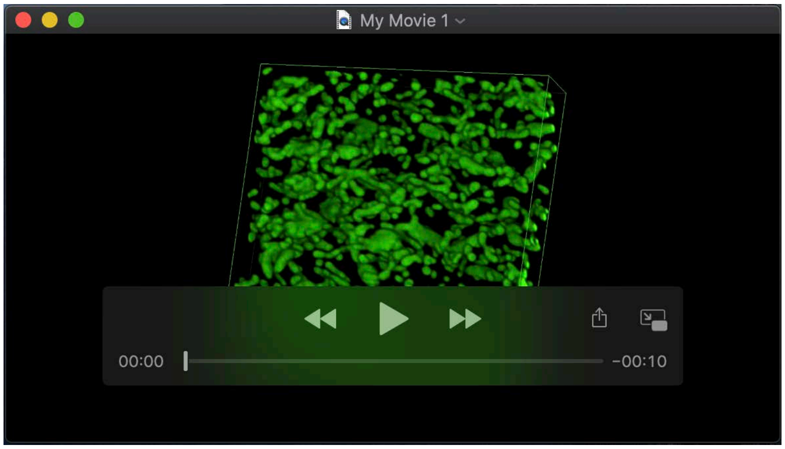

Movie 1. Mitochondria in control salivary gland cells.

3D visualization of mitochondria (marked by mito-GFP, green) in control salivary gland cells (*dpp* > *mito-GFP*).

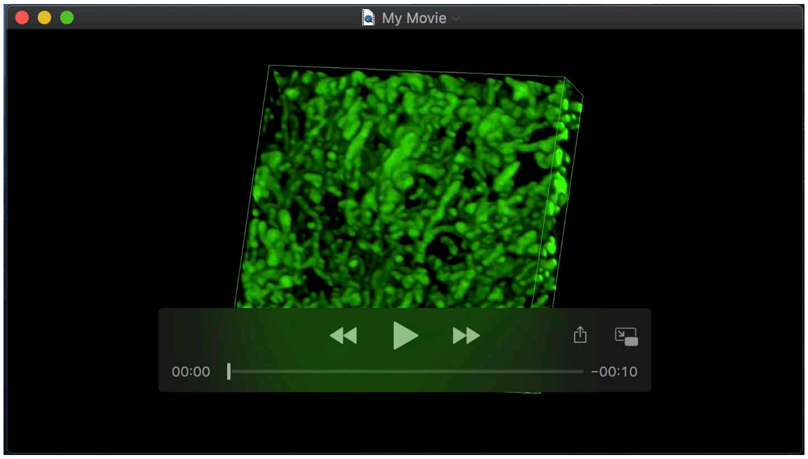

Movie 2. Mitochondria in *hipk*-overexpressing salivary gland cells.

3D visualization of mitochondria (marked by mito-GFP, green) in *hipk*-overexpressing salivary gland cells (*dpp* > *mito-GFP* + *hipk*).

Journal of Cell Science • Supplementary information
