# Supplementary material for: Hyperpolarized mitochondria accumulate in Drosophila Hipk-overexpressing cells to drive tumor-like growth
Source: J Cell Sci. 2020 Dec 9;133(23):jcs250944. doi: 10.1242/jcs.250944 (PMC7746665; doi:10.1242/jcs.250944)
Supplement: Reviewer comments [file joces-133-250944_review_history.pdf]

Hyperpolarized mitochondria accumulate in *Drosophila* Hipk-overexpressing cells to drive tumor-like growth

Kenneth Kin Lam Wong, Jenny Zhe Liao, Claire R.Y. Shih, Nicholas Harden and Esther M. Verheyen

DOI: 10.1242/jcs.250944

Editor: John Heath

Review timeline

Original submission: 30 June 2020
Editorial decision: 14 August 2020
First revision received: 12 October 2020
Accepted: 4 November 2020

Original submission

First decision letter

MS ID#: JOCES/2020/250944

MS TITLE: Functional characterization of the link between mitochondrial metabolism and tumor growth in the Drosophila Hipk tumor model

AUTHORS: Kenneth Kin Lam Wong, Jenny Zhe Liao, Claire R.Y. Shih, Nicholas Harden, and Esther Marianna Verheyen
ARTICLE TYPE: Research Article

We have now reached a decision on the above manuscript.

To see the reviewers' reports and a copy of this decision letter, please go to: https://submit-jcs.biologists.org and click on the 'Manuscripts with Decisions' queue in the Author Area. (Corresponding author only has access to reviews.)

As you will see, the reviewers raise a number of criticisms that prevent me from accepting the paper at this stage. They suggest, however, that a revised version might prove acceptable, if you can address their concerns. If you think that you can deal satisfactorily with the criticisms on revision, I would be pleased to see a revised manuscript. We would then return it to the reviewers.

We are aware that you may currently be unable to access the lab to undertake experimental revisions. If it would be helpful, we encourage you to contact us to discuss your revision in greater detail. Please send us a point-by-point response indicating where you are able to address concerns raised (either experimentally or by changes to the text) and where you will not be able to do so within the normal timeframe of a revision. We will then provide further guidance. Please also note that we are happy to extend revision timeframes as necessary.

Please ensure that you clearly highlight all changes made in the revised manuscript. Please avoid using 'Tracked changes' in Word files as these are lost in PDF conversion.

I should be grateful if you would also provide a point-by-point response detailing how you have dealt with the points raised by the reviewers in the 'Response to Reviewers' box. Please attend to

© 2020. Published by The Company of Biologists under the terms of the Creative Commons Attribution License (https://creativecommons.org/licenses/by/4.0/).

all of the reviewers' comments. If you do not agree with any of their criticisms or suggestions please explain clearly why this is so.

Reviewer 1

Advance summary and potential significance to field

The new interplay of HIPK and mitochondria presented in the paper is significant from the point of basic biology and cancer biology.

Comments for the author

The manuscript by Wong et. al, using a Drosophila model, brings forward some interesting ideas potentially pertaining to tumorigenesis. The manuscript is well written and the experiments have been well planned and logically presented. However, there are some major issues that need to be taken care of to embolden the data presented or modify their conclusions. The following are the specific comments towards improving the manuscript:

1. Are HIPKs overexpressed in cancer tissue? This will justify using overexpressing HIPK in Drosophila to model its role in tumorigenesis. Moreover, care should be taken to not call the Drosophila aberrations as tumors as they differ from the human tumors at fundamental levels.
2. The experimental evidence presented support the title should be modified to "……….mitochondrial energetics…." instead of "mitochondrial metabolism".
3. Their own data show that mitochondrial morphology may not be directly linked to energetics. Therefore, line 99 becomes a weak rationale. The dissociation of morphology and energetics may be due to dependence on abundant glucose, as they demonstrate. This is consistent with PMID: 30910831 and 23562075, which should be cited.
4. It is very important to note that use of Mitotracker red for mitochondrial potential is flawed. TMRE is a more appropriate use, which has been used only in one experiment. Moreover, both mitotracker red and TMRE incorporation is a function of mitochondrial mass and potential. To rule out influence of mass, ratio of TMRE and mitoGFP signal will have to be considered. This becomes more important as the author demonstrate involvement of Myc.
5. Line 168-170: Data not convincing and conclusion far-fetched.
6. There is ample opportunity of quantification of mitochondrial morphology, which has been kept subjective in the paper. If the authors want to draw conclusion about mitochondrial fusion, they need to demonstrate that somehow. Have they tried their images from Airy Scan with the method published in PMID 30910831? It may be possible to obtain the fusion metric from the images of salivary glands. Without any kind of quantification of mitochondrial morphology, the genetic manipulation data remains ambiguous.
7. Although the data presented shows involvement of Myc, it is far-fetched to invoke any feedback loop demonstrated in their previous paper.
8. Fig. 4D remains inconvincing.
9. Line 168-170: conclusion far-fetched.
10. Data presented in Fig. 5 on ROS recapitulates finding from the Banerjee lab? Please cite the appropriately.
11. It is appropriate to say H2O2 is less reactive, as opposed to non-radical. It is still considered as a potent ROS that can be damaging.
12. An important portion of the conclusion drawn relies on supplementary Fig. 7, which is less developed. This experimental arm should be completed and presented with proper quantification as a main figure.
13. The manuscript suffers from quantification at various levels: Mitochondrial morphology (as mentioned above); morphology change of the wing disc has not been described well and marked appropriately in figs, and is quantified only in fig 6; ROS by DHE staining, without which the conclusions are not convincing.
14. Since Fig. 1 shows more striking phenotype in the salivary glands, why isn't that used as the main tissue for the rest of the manuscript, and the data have been largely kept in the supplementary ?
15. The TMRE signal is Fig. 2J does not appear to recapitulate mitoGFP staining 1n. It is very unlikely that those swollen mitochondria maintain tmre to the same level as WT.

© 2020. Published by The Company of Biologists under the terms of the Creative Commons Attribution License (https://creativecommons.org/licenses/by/4.0/).

16. Fig. 4: dashed line in mito-gfp panel missing. panels don't represent the same areas making comparisons difficult.
17. Fig. 6: representative images of sdhrnai and cox5Arnai are missing.
18. A comprehensive schematic of the working model will elevate the value of the findings tremendously.

Reviewer 2

Advance summary and potential significance to field

The manuscript entitled "Functional characterization of the link between mitochondrial metabolism and tumor growth in the Drosophila Hipk tumor model" uses the well-established proto-oncogene Drosophila Homeodomain-interacting protein kinase (Hipk) model to address whether mitochondria are active or not during tumor development.
The authors showed previously in the same model that tumor cells display elevated aerobic glycolysis through a pfk2/dMyc co-stimulation loop. Beyond that findings, the authors show here,on one hand that mitochondria are highly fused and active in tumor cells and that inhibition of complex I is sufficient to suppress Hipk tumor growth. On the other hand, they show that mitochondrial morphology (fusion) is not a key driver for Hipk-mediated tumorigenesis although, fused mitochondria are observed upon Hipk over expression. Finally, the authors investigate the role of ROS in Hipk tumor growth showing that the ROS production need to be kept at a minimum during this process.
The study is very interesting and the model is well established. Through the images, we can appreciate the dramatically increased amount of mitochondria and the enhanced fusion process upon Hipk overexpression. This work adds support to an important role of mitochondrial and Complex I in cancer. Overall, the approaches used in this study are well chosen. However, investigating mitochondria function/respiration only by using MitoTracker Red is rather limited. Also, quantifications are required in almost all panels.

Comments for the author

Figure 1:
➔ It would be more accurate to move supplemental figure 2 in the main figure 1. You could in return move panels g-h of fig1 in the supplementary material.
➔ The control is missing dpp>mito-GFP + marf-RNAi and dpp>mito-GFP + pdsw-RNAi without hipk.
➔ I do not understand the reason to show here the panels m and n? the focus here is fusion/fission. (They could be moved to figure 2 after panel i-j.
➔ In the text (line 125) "In a similar fashion, upon opa1 knockdown, the mitochondria appeared as tiny individual puncta (Fig. 1l), nearly indistinguishable from those in control cells (Fig. 1i). It is surprising that no difference was observed between panel i and l. The absence of OPA1 should induce a hyper fission. Any explanation?
➔ In the text (line 127-128) "Thus, our data suggest that elevated Hipk promotes the formation of highly fused and interconnected mitochondria at least in part through the actions of Marf and Opa1". Same in lines 176-177. I think, it is overstated. The authors should measure Marf and OPA1 expression levels (the isoforms) using Western blot before and after Hipk overexpression. Also, you need to exclude the down regulation of the fission factors.
➔ Upon marf-RNAi treatment (Fig1. k), the whole aspect of the mitochondria is changed. They look swollen. Could you explain that?
➔ Also, it is very clear that not only the mitochondria look more connected but also the amount is much more important upon overexpression of Hipk (the obvious images are shown in fig 1e-f and in supp fig2 i and j). This aspect should be clearly notified and discussed in the text. Moving supp fig2 a-d to the main figure 1 could strengthen this aspect.

Figure 2:

I have major concerns about using only a mitochondrial dye to measure mitochondrial activity.

For example: in lines 145-147, the authors write "In control wing discs, we observed that MitoTracker Red primarily accumulated in myoblasts (MB; the progenitors of adult flight muscles), but not in the

© 2020. Published by The Company of Biologists under the terms of the Creative Commons Attribution License (https://creativecommons.org/licenses/by/4.0/).

DP cells (Fig. 2c, 2e), reflecting differential mitochondrial activities among cell types".
Using only MitoTracker red to conclude about mitochondrial activity is limited, and not true, mostly that the amount of mitochondria in the cell could affect the staining.

1) Since overexpression of Hipk changes the overall structure of the tissue morphology, how can you exclude that the increase in MitoTracker staining observed in Hipk positive cells in not due to the more exposer to the dye? because, as shown in the supp fig 3 and 4, the staining is more important in the periphery. I guess, mostly due to the direct contact of these parts of the tissue to MitoTracker red.

2) Since overexpression of Hipk increases significantly the amount of mitochondria, how can you exclude that the increase in MitoTracker staining is not simply due to increased mitochondrial mass (Supp fig 2 a-d)? You should normalize the red signal to the green signal.

3) In line 155 "the highly convoluted mitochondria in hipk-overexpressing muscle cells display intense MitoTracker Red incorporation (Supp. fig. 5b), showing elevated mitochondrial respiratory activity". Again, I think it is not true. MitoTracker red does not measure mitochondrial respiration. It indicates a sustained mitochondrial membrane potential. You need to use other technics such as high resolution respirometry to measure mitochondrial respiration, or enzymatic assays for the activity of mitochondrial complexes.

4) Line 178 "mitochondria are metabolically active as indicated by the increase in ETC activity ". Overstated again. You can only conclude that the membrane potential is maintained.

Figure 3:

➔ In line 201 : change Fig. 3b'-b" by c' and c''.
➔ MitoTracker red signals need to be quantified (this applies to figure 3 and 4)
➔ The panel b is not at the same magnification as a and c?
➔ There is not need to show here the panel f in suppl. Fig. 6

Figure 4:

➔ Could you explain the difference observed in mitochondrial activity between inhibition of Complex I and V? It was reported that the inhibition of ETC complexes (Not only complex
I) during Drosophila wing disc development leads to robust stimulation of TOR activity (doi: 10.1038/s41598-020-61244-3)? You should consider this publication in the discussion

Figure 5:

➔ ROS signal need to be quantified and confirmed with another staining.

➔ What do you mean with "ROS was minimally produced"? ROS production after Complex I inhibition was reported during Drosophila wing disc development (doi: 10.1038/s41598- 020-61244-3).

➔ Line 269-272 " When pdsw was knocked down in the control background (without Hipk overexpression), ROS was minimally produced (Fig. 5b). Knockdown of ATPsynß in the control background, on the other hand, caused elevated ROS (Fig. 5c). This confirms the model that mitochondrial dysfunction generally brings about ROS production". This is confusing. For me, Knockdown of ATPsynß (fig 5) maintains mitochondrial function! So, why that much ROS production?

Figure 6:
➔ pJNK staining after pdsw-RNAi treatment need to be added in Supp fig7.

First revision

Author response to reviewers' comments

© 2020. Published by The Company of Biologists under the terms of the Creative Commons Attribution License (https://creativecommons.org/licenses/by/4.0/).

MS ID#: JOCES/2020/250944
Original MS TITLE: Functional characterization of the link between mitochondrial metabolism and tumor growth in the Drosophila Hipk tumor model

Responses to Reviewer Comments
We thank the reviewers for their fair and helpful comments and positive assessment of our work. We feel have addressed all reviewer comments. We have incorporated many edits into the text and adjusted figures, as highlighted in the revised manuscript with yellow.

Reviewer 1:
Advance Summary and Potential Significance to Field:
The new interplay of HIPK and mitochondria presented in the paper is significant from the point of basic biology and cancer biology.

Reviewer 1 Comments for the Author:
The manuscript by Wong et. al, using a Drosophila model, brings forward some interesting ideas potentially pertaining to tumorigenesis. The manuscript is well written and the experiments have been well planned and logically presented. However, there are some major issues that need to be taken care of to embolden the data presented or modify their conclusions. The following are the specific comments towards improving the manuscript:

1) Are HIPKs overexpressed in cancer tissue? This will justify using overexpressing HIPK in Drosophila to model its role in tumorigenesis.

The four members of the HIPK family (HIPK1-4) have been implicated in certain cancers (reviewed in Blaquiere and Verheyen, Curr Top Dev Biol. 2017 PMID: 28236976). For instance, elevation of HIPK2 (the most studied member of the family) is associated with the malignancy of pilocytic astrocytomas (Deshmukh *et al.*, Oncogene 2008 PMID: 18408760), cervical carcinogenesis (Al-Beiti and Lu, Aust N Z J Obstet Gynaecol. 2008 PMID: 18532967) and human papillomavirus-positive tonsillar squamous cell carcinomas (Kwon *et al.*, Biomed Res Int. 2017 PMID: 28607924).

We have added this information to the Introduction (please see **Lines 71-74**).

Moreover, care should be taken to not call the Drosophila aberrations as tumors as they differ from the human tumors at fundamental levels.

Larval wing discs, which give rise to adult wings, are extensively used to model human carcinomas (Herranz *et al.*, Curr Top Dev Biol. 2016 PMID: 26970620). Ras^{V12} $scrib^{-/-}$ is one of the well-known fly tumor models (Wu *et al.*, Nature 2010 PMID: 20072127). Our previous work has established a fly tumor model using overexpression of Hipk. This tumor model recapitulates myriad cancer features including tissue overgrowth, loss of epithelial integrity, cell invasion-like behaviors and robust aerobic glycolysis (Blaquiere *et al.*, DMM 2018 PMID: 29208636; Wong *et al.*, eLife 2019 PMID: 31259690).

We understand the reviewer's concern that a fly tumor model is not cancer per se and therefore have made every effort to soften the language in the revised manuscript. We now include 'tumor-like growth' or 'growth aberrations' in place of 'tumors'.

2) The experimental evidence presented support the title should be modified to "……….mitochondrial energetics…." instead of "mitochondrial metabolism".

We have changed our title to 'Hyperpolarized mitochondria accumulate in *Drosophila* Hipk-overexpressing cells to drive tumor-like growth' to reflect the altered mitochondrial energetics observed in Hipk tumor-like cells.

3) Their own data show that mitochondrial morphology may not be directly linked to energetics. Therefore, line 99 becomes a weak rationale. The dissociation of morphology and energetics may be due to dependence on abundant glucose, as they demonstrate. This is consistent with PMID: 30910831 and 23562075, which should be cited.

© 2020. Published by The Company of Biologists under the terms of the Creative Commons Attribution License (https://creativecommons.org/licenses/by/4.0/).

Original line 99: ‘To assess mitochondrial energy metabolism in Hipk-induced neoplasia, we first examined mitochondrial morphology using mito-GFP.’

Thank you for the suggestion. We have rephrased this portion and included the citations accordingly. Please see **Lines 104-108**:

‘In our earlier study, we showed that elevated Hipk increases glucose uptake and glycolytic flux (Wong *et al.*, eLife 2019 PMID: 31259690). Given that mitochondrial metabolism can be influenced by alterations in energy demand and nutrient supply (Liesa and Shirihai, Cell Metab. 2013 PMID: 23562075; Spurlock *et al.*, J Cell Sci. 2019 PMID: 30910831), we were motivated to examine if elevated Hipk leads to any changes regarding mitochondrial dynamics, abundance and energetics.’

4) It is very important to note that use of Mitotracker red for mitochondrial potential is flawed. TMRE is a more appropriate use, which has been used only in one experiment. Moreover, both mitotracker red and TMRE incorporation is a function of mitochondrial mass and potential. To rule out influence of mass, ratio of TMRE and mitoGFP signal will have to be considered. This becomes more important as the author demonstrate involvement of Myc.

We are aware that some MitoTracker dyes (e.g. MitoTracker™ Green FM, Invitrogen, #M7514) label mitochondria independently of the membrane potential and are used as a mitochondrial mass marker. However, the dye we used (MitoTracker™ Red CMXRos, Invitrogen, #M7512) labels mitochondria in a membrane potential-dependent manner, which is similar to TMRM or TMRE. The efficacy of MitoTracker Red for measurement of membrane potential has been demonstrated by others (e.g. PMID: 15382028, 26330624), and the dye has been used by many to assess mitochondrial energetics (e.g. PMID: 30160596, 25918408, 29157562, 32157127).

We agree that the dye incorporation depends on both mitochondrial mass and membrane potential. Therefore, we now include quantifications of the ratio of MitoTracker Red to mito-GFP signal intensities to rule out the influence of mitochondrial mass **(Figs. 2I’’, 3I’’’, S5C)**.

5) Line 168-170: Data not convincing and conclusion far-fetched.

Original line 168-170: ‘We found that genetic inhibition of Complex V did not significantly affect TMRM accumulation in hipk-overexpressing cells (Fig. 2j-k), implying that elevated Hipk sustains negative Δψm by stimulating the ETC activity but not by the reversed function of Complex V.’

We have rephrased our description and softened our language regarding to the result interpretation:

‘We found that *pdsw-RNAi*, but not *ATPsynβ-RNAi*, markedly suppressed the TMRM accumulation in *hipk*-overexpressing cells **(Fig. 2L-N)**, inferring that Complex I is primarily required for sustaining the membrane potential. ... Thus, we favor the model that Complexes I/III/IV in the ETC sustain the membrane potential in cells with elevated Hipk over the model that the respiratory chain is compromised and Complex V is functioning in reverse to sustain the potential.’ **(Lines 209-216)**

We also provide a more explicit rationale for the experimental design prior to presenting the results to help with the interpretation.

‘Mitochondrial membrane potential is established by pumping protons from the mitochondrial matrix to the intermembrane space. This can be achieved either by the ETC Complexes I/III/IV of the electron transport chain (ETC) in healthy, functional mitochondria **(Fig. 2A)**, or by the reversal of Complex V (ATP synthase) function (which occurs when the normal operation of the ETC is compromised) **(Fig. 2A’)** (Zorova *et al.*, Anal Biochem. 2018 PMID: 28711444). To identify the cause of membrane hyperpolarization in *hipk*-overexpressing cells, we used RNAi to individually knock down *pdsw* (or *ND- pdsw*) and *ATPsynβ*, which are genes that encode a Complex I subunit and a Complex V subunit, respectively **(Fig. 2A)**. If the Δψm is sustained by Complexes I/III/IV, *pdsw* knockdown and *ATPsynβ* knockdown will suppress and maintain TMRM accumulation, respectively. On the contrary, if the Δψm is sustained by the reversal of Complex V function, *pdsw* knockdown

© 2020. Published by The Company of Biologists under the terms of the Creative Commons Attribution License (https://creativecommons.org/licenses/by/4.0/).

will have little effect on TMRM staining whereas *ATPsynB* knockdown will reduce the staining.' **(Lines 198-209)**

6) There is ample opportunity of quantification of mitochondrial morphology, which has been kept subjective in the paper. If the authors want to draw conclusion about mitochondrial fusion, they need to demonstrate that somehow. Have they tried their images from Airy Scan with the method published in PMID 30910831? It may be possible to obtain the fusion metric from the images of salivary glands. Without any kind of quantification of mitochondrial morphology, the genetic manipulation data remains ambiguous.

We thank the reviewer for the suggestion. We now include the quantification of mitochondrial morphology in **Figs. 1, 3, S2**. Since we have been using the MiNA toolset (Valente *et al*., Acta Histochem. 2017 PMID: 28314612), we have decided to continue using it to quantify multiple features of mitochondrial morphology. We also mention additional image analysis tools (e.g. MitoGraph (PMID: 25640425) and Mito-SinCe^2 (PMID: 30910831)) for measuring the morphology quantitatively (Please see **Lines 111- 115**).

7) Although the data presented shows involvement of Myc, it is far-fetched to invoke any feedback loop demonstrated in their previous paper.

When Myc and Pfk2 were knocked down, the mitochondrial changes (fusion, mass, membrane hyperpolarization) caused by elevated Hipk were largely suppressed **(Fig. 3A-D)**. We have rephrased the data interpretation as 'our data suggest that the changes in mitochondrial morphology, abundance and energetics in Hipk tumor-like cells require Myc and Pfk2' (Please see **Line 239-241**).

We have excluded the possible involvement of the Myc-aerobic glycolysis feedback loop from the Results section and only speculate in the Discussion section that the changes in the mitochondrial profile could possibly be driven downstream of the loop.

8) Fig. 4D remains unconvincing.

We now move the original Fig. 4D to new **Fig. 4G** (*dpp>mito-GFP + hipk + pdsw-RNAi*).

For better comparison, we now show all the merged and individual channels (GFP in green showing the transgene-expressing Dpp domain and DNA in blue showing the tissue morphology). Compared to *hipk*-overexpressing wing discs, *pdsw-RNAi* co-expression largely suppressed the distorted tissue morphology (compare **Fig. 4G** with **Fig. 4B**).

Additionally, we now provide schematic diagrams to show the distorted folding caused by elevated Hipk in wing discs (please see **Fig. 1A''', 1C'''**).

9) Line 168-170: conclusion far-fetched.

Please see the response to comment #5.

10) Data presented in Fig. 5 on ROS recapitulates finding from the Banerjee lab? Please cite the appropriately.

Our data are in line with previous findings from Banerjee's lab showing that Complex I disruption increases ROS production. The corresponding citation has been included (Please see **Lines 302-303**).

11) It is appropriate to say H2O2 is less reactive, as opposed to non-radical. It is still considered as a potent ROS that can be damaging.

We now replace the phase 'less damaging, non-radical' by 'less reactive' **(Line 329)**.

12) An important portion of the conclusion drawn relies on supplementary Fig. 7, which is less

© 2020. Published by The Company of Biologists under the terms of the Creative Commons Attribution License (https://creativecommons.org/licenses/by/4.0/).

developed. This experimental arm should be completed and presented with proper quantification as a main figure.

We have expanded this experimental arm by examining the pJNK and MMP1 levels of control discs (*dpp > GFP*), *hipk*-overexpressing discs with *pdsw* knockdown (*dpp > GFP + hipk + pdsw-RNAi*) and *ATPsynβ* knockdown discs (*dpp > GFP + ATPsynβ-RNAi*) **(Fig. 7)**.

Quantifications of the relative pJNK and MMP1 levels are included **(Fig. 7G-G')**.

13) The manuscript suffers from quantification at various levels: Mitochondrial morphology (as mentioned above); morphology change of the wing disc has not been described well and marked appropriately in figs, and is quantified only in fig 6; ROS by DHE staining, without which the conclusions are not convincing.

We now provide quantifications of mitochondrial morphology **(Fig. 1, 3, S2)**, Mitotracker Red to mito- GFP ratio **(Figs. 2, 3, S5)**, mean TMRM intensity **(Fig. 2)**, relative ROS levels **(Fig. 5)**, wing disc morphology **(Fig. 6)** and relative pJNK and MMP1 levels **(Fig. 7)**.

We also add schematic diagrams to mark the morphology change of wing discs upon *hipk* overexpression (Fig. 1A''', 1C''').

14) Since Fig. 1 shows more striking phenotype in the salivary glands, why isn't that used as the main tissue for the rest of the manuscript, and the data have been largely kept in the supplementary ?

In this study, we primarily used wing discs because overexpression of *hipk* in wing discs induce tissue overgrowth and other abnormalities reminiscent of tumor progression (Blaquiere *et al.*, DMM 2018 PMID: 29208636). However, we are aware the wing disc epithelial cells are very small in size such that the changes in mitochondrial morphology are not clear enough. We therefore use additional tissues, salivary glands and muscle walls, because their large cell size facilitates the visualization of cellular organelles. Salivary glands and muscle walls, on the contrary, do not display tumor-like growth upon *hipk* overexpression.

Nonetheless, we showed that elevated Hipk exerted comparable effects on mitochondrial morphology, abundance and energetics in both wing disc and salivary gland epithelial cells **(Figs. 1, 2, S2)**. In larval muscle cells, elevation of Hipk only led to increases in mitochondrial branching and membrane potential, but not mitochondrial length or mass **(Fig. S2)**, likely because control muscle cells (without *hipk* overexpression) are already fused and abundant (unlike epithelial cells).

15) The TMRE signal is Fig. 2J does not appear to recapitulate mitoGFP staining 1n. It is very unlikely that those swollen mitochondria maintain tmre to the same level as WT.

TMRM signal in the original Fig. 2J (new **Fig. 2M** *dpp > hipk + ATPsynβ-RNAi*) stains mitochondria in a membrane potential-dependent manner, whereas mito-GFP in the original Fig. 1n (moved to **Fig. S2K**) labels mitochondria independently of the potential. The differential staining suggests that the mitochondria took up TMRM at different rates. In other words, the mitochondria were polarized to a varying extent. Consistent with this notion, an earlier study shows that even in a single mitochondrion, individual cristae display differential membrane potentials (PMID: 31609012). Nonetheless, currently we do not have an explanation why knockdown of *ATPsynβ* will cause such effect.

16) Fig. 4: dashed line in mito-gfp panel missing. panels don't represent the same areas, making comparisons difficult.

We apologize for the confusion about this panel. For easier comparison, we now focus on the transgene- expressing disc proper cells only **(Fig. 3)**.

17) Fig. 6: representative images of sdhrnai and cox5Arnai are missing.

We now include the wing disc images of *dpp > hipk + sdhC-RNAi* and *dpp > hipk + cox5A-RNAi*

© 2020. Published by The Company of Biologists under the terms of the Creative Commons Attribution License (https://creativecommons.org/licenses/by/4.0/).

without or with *SOD1*/*SOD2*/*catalase* overexpression in **Fig. 6O-V**.

18) A comprehensive schematic of the working model will elevate the value of the findings tremendously.

Thank you for the constructive suggestion. We now include a schematic diagram depicting our working model **(Fig. 7H**; please see **Lines 373-377)**.

Reviewer 2
The manuscript entitled "Functional characterization of the link between mitochondrial metabolism and tumor growth in the Drosophila Hipk tumor model" uses the well-established proto-oncogene Drosophila Homeodomain-interacting protein kinase (Hipk) model to address whether mitochondria are active or not during tumor development.

The authors showed previously in the same model that tumor cells display elevated aerobic glycolysis through a pfk2/dMyc co-stimulation loop. Beyond that findings, the authors show here, on one hand that mitochondria are highly fused and active in tumor cells and that inhibition of complex I is sufficient to suppress Hipk tumor growth. On the other hand, they show that mitochondrial morphology (fusion) is not a key driver for Hipk-mediated tumorigenesis although, fused mitochondria are observed upon Hipk over expression. Finally, the authors investigate the role of ROS in Hipk tumor growth showing that the ROS production need to be kept at a minimum during this process.

The study is very interesting and the model is well established. Through the images, we can appreciate the dramatically increased amount of mitochondria and the enhanced fusion process upon Hipk overexpression. This work adds support to an important role of mitochondrial and Complex I in cancer.
Overall, the approaches used in this study are well chosen. However, investigating mitochondria function/respiration only by using MitoTracker Red is rather limited. Also, quantifications are required in almost all panels.

Figure 1:

1.1 It would be more accurate to move supplemental figure 2 in the main figure 1. You could in return move panels g-h of fig1 in the supplementary material.

Thank you for the suggestion. We now include the quantification of mitochondrial morphology in **Fig. 1**, and move the original Fig. 1g-h to **Fig. S2D-E**.

1.2 The control is missing dpp>mito-GFP + marf-RNAi and dpp>mito-GFP + pdsw-RNAi without hipk.

We now add the proper controls (*marf-RNAi*, *opa1-RNAi*, *pdsw-RNAi* and *ATPsynB-RNAi*) to **Fig. S2F-I**.

1.3 I do not understand the reason to show here the panels m and n? the focus here is fusion/fission. (They could be moved to figure 2 after panel i-j.

We regret the confusion. To demonstrate that the suppression of TMRM accumulation in *hipk*-overexpressing cells by *pdsw-RNAi* is not caused by loss of mitochondria, we used mito-GFP to reveal the mitochondrial mass and found that mitochondria remained abundant in *dpp > mito-GFP + hipk + pdsw- RNAi* cells. We now move the original Fig. 1m-n to **Fig. S2J-K**.

1.4 In the text (line 125) "In a similar fashion, upon opa1 knockdown, the mitochondria appeared as tiny individual puncta (Fig. 1l), nearly indistinguishable from those in control cells (Fig. 1i). It is surprising that no difference was observed between panel i and l. The absence of OPA1 should induce a hyper fission. Any explanation?

We regret that we were not clear enough in our description. Indeed, we observed a hyper-fission phenotype in the *hipk*-overexpressing cells when *opa1* was knocked down. In the control cells, both

© 2020. Published by The Company of Biologists under the terms of the Creative Commons Attribution License (https://creativecommons.org/licenses/by/4.0/).

rod- shaped and dot-like mitochondria were seen **(Fig. 1F)**. On the contrary, in *hipk* + *opa1-RNAi* co- expressing cells, the mitochondria primarily appeared as tiny individual punctate, and rod-shaped mitochondria were rarely found **(Fig. 1I)**.

We now replaced the phrase 'nearly indistinguishable from those in control cells' by 'showing a robust fission phenotype' (please see **Line 146-147**).

1.5 In the text (line 127-128) "Thus, our data suggest that elevated Hipk promotes the formation of highly fused and interconnected mitochondria at least in part through the actions of Marf and Opa1". Same in lines 176-177. I think, it is overstated. The authors should measure Marf and OPA1 expression levels (the isoforms) using Western blot before and after Hipk overexpression. Also, you need to exclude the down regulation of the fission factors.

We are intrigued by the reviewer's suggestion about the regulation of fusion and fission regulators. As mentioned in the Discussion, the fusion regulator genes, *marf* and *opa1*are target genes of Yki (Nagaraj et al., 2012; Neto-Silva et al., 2010), and Hipk is a positive regulator of Yki (Chen and Verheyen, 2012; Poon et al., 2012). Thus, it is conceivable that elevated Hipk promotes mitochondrial fusion through upregulation of *marf* and *opa1*. To test this idea, we did qRT-PCR to determine the relative mRNA levels of *marf* and *opa1* upon *hipk* expression. Indeed, we found that *marf* and *opa1* transcription was significantly induced in *hipk*-overexpressing discs. However, currently we do not have antibodies to test their protein levels. [We have removed a figure which was provided for the referees in confidence]

We also tested if the fission regulator Drp1 is downregulated upon *hipk* overexpression using antibodies against Drp1 and pDrp1-S616. To our surprise, we noticed that there were mild increases in the levels of both Drp1 and pDrp1-S616 (an active form). Thus, it is tempting to speculate that both fusion and fission regulators are upregulated in Hipk tumor-like cells, giving a net result of mitochondrial fusion. Supporting our speculation, we observed that *pdsw* knockdown further enhanced the fusion phenotype in *hipk*-overexpressing cells, possibly in an attempt to increase mitochondrial function to meet the energy demand. [We have removed a figure which was provided for the referees in confidence]

Nevertheless, we feel that these results are still preliminary at the current stage. Our revised manuscript focuses on the characterization of the mitochondrial profile of the Hipk tumor-like cells. Further studies are required to elucidate the cause(s) of the metabolic shift in molecular detail.

1.6 Upon marf-RNAi treatment (Fig1. k), the whole aspect of the mitochondria is changed. They look swollen. Could you explain that?

Indeed, we observed that *marf-RNAi* led to the formation of large, spherical mitochondrial aggregates (regardless of *hipk* expression levels) **(Figs. 1H, S2F)**, whereas such phenotype was not seen when *opa1* was knocked down. Similar observations have been made by others in neuronal cells (PMID: 29742430), reflecting the fundamental difference in the functions of Marf (mediating the fusion of outer mitochondrial membrane) and Opa1 (mediating the fusion of inner mitochondrial membrane).

We speculate that when Marf is deficient, fusion of the inner membrane (mediated by Opa1) continues, forming a large matrix compartment wrapped around by the outer membrane. On the other hand, when Opa1 is deficient, the matrix compartments cannot fuse together, forming tiny mitochondrial particles.

Unfortunately, we do not have any evidence to support our speculations. Nonetheless, our observations highlight the requirement of both Marf and Opa1 in mitochondrial fusion in *hipk*-overexpressing cells.

1.7 Also, it is very clear that not only the mitochondria look more connected but also the amount is much more important upon overexpression of Hipk (the obvious images are shown in fig 1e-f and in supp fig2 i and j). This aspect should be clearly notified and discussed in the text. Moving supp fig2 a-d to the main figure 1 could strengthen this aspect.

© 2020. Published by The Company of Biologists under the terms of the Creative Commons Attribution License (https://creativecommons.org/licenses/by/4.0/).

Thanks for the suggestion. We now move the quantification of mitochondrial morphology and mass to **Fig. 1** and highlight our observation that elevated Hipk led to a significant increase in mitochondrial abundance in the text (e.g. see **Lines 115-118, 131-133**).

Figure 2:

I have major concerns about using only a mitochondrial dye to measure mitochondrial activity.

For example: in lines 145-147, the authors write “In control wing discs, we observed that MitoTracker Red primarily accumulated in myoblasts (MB; the progenitors of adult flight muscles), but not in the DP cells (Fig. 2c, 2e), reflecting differential mitochondrial activities among cell types”.

Using only MitoTracker red to conclude about mitochondrial activity is limited, and not true, mostly that the amount of mitochondria in the cell could affect the staining.

We concur with the reviewer that the MitoTracker Red staining depends on both mitochondrial mass and membrane potential. We have therefore removed the phrase ‘reflecting differential mitochondrial activities among cell types’ from the manuscript.

2.1 Since overexpression of Hipk changes the overall structure of the tissue morphology, how can you exclude that the increase in MitoTracker staining observed in Hipk positive cells in not due to the more exposer to the dye? because, as shown in the supp fig 3 and 4, the staining is more important in the periphery. I guess, mostly due to the direct contact of these parts of the tissue to MitoTracker red.

We agree that the MitoTracker Red dye is poor at penetrating into deeper tissues. Therefore, we focus on the basal side of wing discs where the disc proper cells are more exposed to the dye (whereas, on the apical side, the access to the dye is blocked by the peripodial membrane). More importantly, given that the myoblasts have robust MitoTracker Red staining, we have been using the MitoTracker Red staining in myoblasts as an internal reference in each wing disc to ensure that all the disc proper cells we captured had been exposed to comparable amounts of the dye **(Lines 178-181)**.

2.2 Since overexpression of Hipk increases significantly the amount of mitochondria, how can you exclude that the increase in MitoTracker staining is not simply due to increased mitochondrial mass (Supp fig 2 a-d)? You should normalize the red signal to the green signal.

We agree with the reviewers’ comment on the dependence of MitoTracker staining on both mitochondrial abundance and membrane potential. We now include the normalization and assess the membrane potential as a ratio of MitoTracker Red to mito-GFP signal intensities **(Figs. 2, 3, S5)**.

2.3 In line 155 “the highly convoluted mitochondria in hipk-overexpressing muscle cells display intense MitoTracker Red incorporation (Supp. fig. 5b), showing elevated mitochondrial respiratory activity“. Again, I think it is not true. MitoTracker red does not measure mitochondrial respiration. It indicates a sustained mitochondrial membrane potential. You need to use other technics such as high resolution respirometry to measure mitochondrial respiration, or enzymatic assays for the activity of mitochondrial complexes.

We have replaced the phrase ‘elevated mitochondrial respiratory activity’ by ‘sustained membrane potential’ (please see **Line 189**) for the precise description of the results.

We agree with the reviewer that the respiratory activity cannot be determined by MitoTracker Red staining, and that additional techniques are required for the comprehensive profiling of mitochondrial metabolism, which we may pursue in future studies.

2.4 Line 178 “mitochondria are metabolically active as indicated by the increase in ETC activity “. Overstated again. You can only conclude that the membrane potential is maintained.

We thank the reviewer for pointing out the issue and have replaced the phrase by ‘hyperpolarized mitochondria’ **(Line 220)**.

© 2020. Published by The Company of Biologists under the terms of the Creative Commons Attribution License (https://creativecommons.org/licenses/by/4.0/).

Figure 3:

3.1 In line 201 : change Fig. 3b'-b" by c' and c''.

We have now replaced the original Fig. 3 with a new one and changed the text accordingly.

3.2 MitoTracker red signals need to be quantified (this applies to figure 3 and 4)

We now include quantification of the ratio of MitoTracker Red to mito-GFP signal intensities in **Figs. 2, 3 S5**.

3.3 The panel b is not at the same magnification as a and c?

Thank you for pointing out the mistake. The scale bars are now provided in the new **Fig. 4**.

3.4 There is no need to show here the panel f in suppl. Fig. 6

The panel in Fig. S6F shows that in salivary gland cells, the mitochondrial fusion caused by elevated Hipk could not be suppressed *pdsw-RNAi* even though the membrane potential is substantially reduced, which corroborates the wing disc analysis **(Fig. 3F)**. Therefore, we feel that the inclusion of the salivary gland data in the supplemental material rather than removal is needed to complement the wing disc analysis.

Figure 4:

4.1 Could you explain the difference observed in mitochondrial activity between inhibition of Complex I and V? It was reported that the inhibition of ETC complexes (Not only complex I) during Drosophila wing disc development leads to robust stimulation of TOR activity (doi: 10.1038/s41598-020-61244-3)? You should consider this publication in the discussion

Thank you for asking for clarification. For details, please see the response to comment #5 raised by Reviewer 1. Briefly, inhibition of Complex I will reduce proton pumping from the matrix to the intermembrane space, and thus we expect to observe a reduction in the Mitotracker Red/TMRM staining, which is indicative of membrane depolarization. The effects on membrane potential by Complex V inhibition can vary because Complex V can function in both directions, i.e. (1) allowing protons to get into the matrix from the intermembrane space (depolarization) and (2) pumping protons from the matrix to the intermembrane space (hyperpolarization). Inhibition of Complex V will lower the membrane potential in the latter case, but not in the former.

We have now included the suggested publication in the Discussion section to highlight the important roles of mitochondrial metabolism in physiological tissue development and in various tumor models (please see **Lines 384-386**).

Figure 5:

5.1 ROS signal need to be quantified and confirmed with another staining.

The quantification of the relative ROS levels is now shown in **Fig. 5G**.

In response to reviewer's suggestion, we tried another dye, 2',7'-Dichlorofluorescin diacetate (DCFDA), to test the ROS levels. Intriguingly, we observed no noticeable changes in the DCFDA staining in the *hipk*-overexpressing discs or *pdsw/ATPsynB-RNAi* discs. To demonstrate that the dye is working properly, we induced physical damages to control wing discs by poking the discs with dissecting forceps before the dye incubation. Notably, the damage caused robust DCFDA staining, which is indicative of elevated ROS and consistent with a previous study (PMID 28753614). [We have removed a figure which was provided for the referees in confidence]

Our data might suggest that DCFDA is less sensitive than DHE to probe for ROS. Alternatively, given that DHE is primarily oxidized by superoxide while DCFDA is oxidized by peroxides (e.g. H_2O_2), peroxynitrite and hydroxyl radical (PMID: 9701053, 2159514, doi:10.1038/nprot.2008.23), it is

© 2020. Published by The Company of Biologists under the terms of the Creative Commons Attribution License (https://creativecommons.org/licenses/by/4.0/).

possible that superoxide is the primary ROS produced upon *pdsw/ATPsynβ* knockdown. Nonetheless, we deem that the examination of type-specific ROS would be beyond the scope of the current study, and thus the DCFDA staining is not included in the revised manuscript.

5.2 What do you mean with “ROS was minimally produced”? ROS production after Complex I inhibition was reported during Drosophila wing disc development (doi: 10.1038/s41598- 020-61244-3).

We apologize for the confusion caused by the inappropriate description. We were comparing the ROS levels in *pdsw-RNAi* and *ATPsynβ-RNAi* discs, and our data show that the relative ROS levels in *pdsw- RNAi* wing discs were lower than those in *ATPsynβ-RNAi* ones (compare **Fig. 5B** with **Fig. 5C**).

We now provide a more precise description of our results:

‘When *pdsw* was knocked down in the control background (without *hipk* overexpression), we observed 10-20% increase in ROS levels in the knockdown cells relative to the neighboring control cells **(Fig. 5B, 5G)**, consistent with a previous study showing elevated ROS production upon Complex I inhibition (Perez-Gomez et al., 2020). Knockdown of *ATPsynβ* increased the relative ROS levels by 30-40% **(Fig. 5C, 5G)**’ (please see **Line 306-310**)

5.3 Line 269-272 “ When pdsw was knocked down in the control background (without Hipk overexpression), ROS was minimally produced (Fig. 5b). Knockdown of ATPsynβ in the control background, on the other hand, caused elevated ROS (Fig. 5c). This confirms the model that mitochondrial dysfunction generally brings about ROS production”. This is confusing. For me, Knockdown of ATPsynβ (fig 5) maintains mitochondrial function! So, why that much ROS production?

We regret for the confusion caused. Normally, ATP synthase (Complex V) couples the proton motive force to the synthesis of ATP. As a result, the membrane potential will be diminished as protons flow back from the intermembrane space to the matrix. Inhibition of ATP synthase will stop channeling the protons and thus the membrane potential continues to build up by the ETC Complexes (I-IV) until a threshold is reached (when the proton gradient is too large that Complexes I/III/IV can no longer pumps protons; i.e. the ETC is compromised).

Our data show that genetic inhibition of ATP synthase by *ATPsynβ-RNAi* sustained the membrane hyperpolarization caused by *hipk* overexpression, inferring that the potential is maintained by the ETC Complexes. This is consistent with earlier studies showing that inhibition of Complex V (by overexpression of Inhibitory Factor 1) promotes membrane polarization and the subsequent ROS production (Formentini *et al.*, Mol Cell. 2012 PMID: 22342343).

Figure 6:

6.1 pJNK staining after pdsw-RNAi treatment need to be added in Supp fig7.

In response to reviewer’s suggestion, we performed the pJNK and MMP1 staining of *dpp > GFP + hipk*
+ pdsw-RNAi wing discs and the data are shown in new **Fig. 7**.

<u>Second decision letter</u>

MS ID#: JOCES/2020/250944

MS TITLE: Hyperpolarized mitochondria accumulate in Drosophila Hipk-overexpressing cells to drive tumor-like growth

AUTHORS: Kenneth Kin Lam Wong, Jenny Zhe Liao, Claire R.Y. Shih, Nicholas Harden, and Esther Marianna Verheyen

© 2020. Published by The Company of Biologists under the terms of the Creative Commons Attribution License (https://creativecommons.org/licenses/by/4.0/).

ARTICLE TYPE: Research Article

I am happy to tell you that your manuscript has been accepted for publication in Journal of Cell Science, pending standard ethics checks.

Reviewer 1

Advance summary and potential significance to field

The authors have addressed all the major concerns satisfactorily. Therefore publication is recommended.

Comments for the author

None

Reviewer 2

Advance summary and potential significance to field

The authors show in the Drosophila Hipk overexpression-tumor model that mitochondria are highly active and fused in tumor cells and inhibition of complex I, and not inhibition of the fusion actors, was sufficient to suppress tumor growth. Thus, this study confirms that the Warburg effect doesn't apply to all tumors and, instead, it appears that the biological role of mitochondria, might be, is tumor-type specific.

Comments for the author

In the revised manuscript, the authors properly addressed all concerns that I raised. Now, the manuscript benefit from the added quantifications in the figures and the clarifications in the text to enhance its impact on our understating of the controversial role of mitochondria during cancer deployment. The manuscript is now appropriate for publication in Journal of Cell Science.

© 2020. Published by The Company of Biologists under the terms of the Creative Commons Attribution License (https://creativecommons.org/licenses/by/4.0/).
